# Single‐Cell Fluorescence Imaging Reveals Heterogeneity in Senescence Biomarkers and Identifies Rapamycin‐Responsive Sub‐Populations

**DOI:** 10.1111/acel.70209

**Published:** 2025-09-03

**Authors:** Vijayraghavan Seshadri, Charmaine Chng, Joel Tyler, Cestarangga Adikerta, Kaveh Baghaei, Yan Wang, Nuri Gueven, Sharon Ricardo, Iman Azimi

**Affiliations:** ^1^ Monash Biomedicine Discovery Institute, Department of Pharmacology Monash University Clayton Victoria Australia; ^2^ School of Pharmacy and Pharmacology, College of Health and Medicine University of Tasmania Hobart Tasmania Australia

## Abstract

Cellular senescence is a state of irreversible cell cycle arrest accompanied by a distinctive inflammatory secretory profile known as the senescence‐associated secretory phenotype (SASP). While various biomarkers, such as senescence‐associated beta‐galactosidase (SA‐βgal), EdU incorporation, p21 and p16, are used to identify senescent cells, no single biomarker universally defines cellular senescence and current methods often fail to address heterogeneity in biomarker expression levels. This study leverages single‐cell fluorescence imaging to assess multiple senescence markers including SA‐βgal enzymatic activity, p21 and IL‐6 expression and nuclear and cell area in chemotherapy‐induced (mitomycin C) and oxidative stress‐induced (D‐galactose) senescence models in human fibroblasts. Our findings reveal significant heterogeneity in SA‐βgal activity and distinct sub‐populations within senescent cells. Nuclear and cell area measurements emerged as robust indicators of cellular senescence, displaying similar variability across individual cells. Importantly, we identified specific nuclear area sub‐populations that strongly correlate with IL‐6 expression levels, demonstrating a relationship between the heterogeneous expression of senescence biomarkers and the SASP. To address this heterogeneity, we introduced an induction threshold method to more accurately quantify the percentage of cells expressing senescence biomarkers. Furthermore, in both senescence models, we observed that rapamycin, a well‐known senomorphic agent, selectively targets specific biomarker‐expressing sub‐populations. This study underscores the value of assessing cellular heterogeneity in senescence research and provides an improved approach for analysing senescence markers in diverse cellular contexts.

## Graphical abstract

Cellular senescence induced by distinct stressors generated heterogeneous populations with variable expression of canonical biomarkers. Rapamycin showed selective senomorphic effects on specific subpopulations, while the senolytic agent ABT‐263 effectively eliminated all senescent cells, regardless of biomarker heterogeneity.

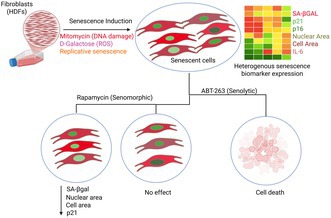

## 1 Introduction

Cellular senescence is closely associated with organismal ageing and becomes more prevalent with age, especially in organs affected by age‐related diseases (Hayflick and Moorhead 1961; Yousefzadeh et al. 2021). Senescence involves irreversible growth arrest due to natural ageing or in response to different forms of stress. It is characterised by a heightened secretory phenotype and resistance to apoptosis (Shay and Wright 2000; Herranz and Gil 2018; Childs et al. 2014). Senescence impacts many different biological processes, including tissue homeostasis, embryonic development, wound healing, immune responses, cell clearance and cancer (Herranz and Gil 2018; Childs et al. 2014).

Proliferative senescence was first reported by Hayflick and Moorhead in 1961, who observed that normal cultured human fibroblasts have a finite capacity for cell division under optimal in vitro conditions, eventually entering a stage of irreversible growth arrest (Hayflick and Moorhead 1961; Shay and Wright 2000). In addition to natural ageing, cellular senescence can be accelerated by different stressors, such as oxidative stress, oncogene activation and acute DNA damage caused by chemotherapy or radiotherapy (Lee and Lee 2019; Gorgoulis et al. 2019). These stressors induce DNA damage that cells cannot fully repair, activating the DNA damage response signalling pathway and leading to senescence (Lee and Lee 2019; Gorgoulis et al. 2019; Domen et al. 2022; McHugh and Gil 2018).

Despite their non‐proliferative status, senescent cells remain metabolically active and adopt a pro‐inflammatory state, releasing cytokines, chemokines, growth factors and proteases. This collective pro‐inflammatory state is termed the senescence‐associated secretory phenotype (SASP) (Di Micco et al. 2021; Coppé et al. 2010). Through the SASP, senescent cells communicate with the immune system to either facilitate their clearance or support tissue regeneration. However, this process can also induce senescence in neighbouring healthy cells, contributing to tissue degeneration.

The effects of senescent cells within tissues can be both positive and negative, depending on whether these cells are transient or persistent. Acute senescence plays a beneficial role in tumour suppression, wound healing and tissue homeostasis, as the SASP signals the immune system to clear senescent cells and promotes the repair of damaged tissue (Yousefzadeh et al. 2021; Davalos et al. 2010). Conversely, with age, the accumulation of senescent cells increases due to the declining efficiency of the ageing immune system to remove them (Coppé et al. 2010; Davalos et al. 2010). This accumulation disrupts tissue structure and function and leads to further induction of senescence. Over time, this build‐up, referred to as chronic senescence, results in the persistent release of SASP factors into the microenvironment (Coppé et al. 2010; Davalos et al. 2010). Consequently, the chronic inflammatory environment induced by these persistent senescent cells becomes harmful, contributing to the progression of various age‐related diseases, such as osteoarthritis, atherosclerosis, neurodegenerative disorders and tumorigenesis (Yousefzadeh et al. 2021; Amaya‐Montoya et al. 2020). Thus, the paradoxical nature of senescence provides short‐term benefits but also poses long‐term liabilities as senescent cells accumulate over time.

Senescent cells exhibit distinct characteristics that differentiate them from non‐senescent cells, providing opportunities for targeted therapeutic interventions. These cells are characterised by specific marker proteins, including the increased expression of cyclin‐dependent kinase inhibitors p16, p21 and TP53, which are induced to halt cell proliferation (Domen et al. 2022; McHugh and Gil 2018; Schwartz and Shah 2005). Additionally, senescent cells display unique morphological features, such as enlarged and flattened cellular shapes, irregular and enlarged nuclei and heightened enzymatic activity of the lysosomal enzyme senescence‐associated beta‐galactosidase (SA‐β‐gal), among others (McHugh and Gil 2018).

While these features are commonly associated with senescence, they are not exclusive indicators. For instance, the cyclin‐dependent kinase inhibitor p21 is not universally upregulated in all senescent cells, making it an unreliable marker when used alone (He and Sharpless 2017). Similarly, p53, known for its role in cellular apoptosis, does not definitively distinguish between senescent and apoptotic cells (Amaral et al. 2010). Furthermore, SA‐β‐gal, a widely used senescence marker, is also expressed by other cell types, such as macrophages (Dimri et al. 1995; de Mera‐Rodríguez et al. 2021). Conditions like high cell density or exposure to hydrogen peroxide can also trigger SA‐β‐gal activity, leading to false positives in senescence detection (Yang and Hu 2005).

The diversity of senescent cell traits and the lack of a single universal marker necessitate a comprehensive approach that combines multiple senescence biomarkers for precise and reliable identification and quantification of senescent cells. However, this integration presents detection challenges. Traditional methods, such as Western blotting, immunofluorescence and flow cytometry, often lack the specificity and sensitivity required. While Western blotting can detect specific protein levels, it falls short in providing information on cellular morphology or the spatial distribution of senescence markers and does not capture single‐cell heterogeneity in biomarker expression. Immunofluorescence and flow cytometry offer more detailed spatial and quantitative data but struggle due to the nonspecific nature of many senescence markers and the heterogeneity among senescent cell populations. Furthermore, these methods typically involve fixed cells, limiting the ability to study live cell dynamics. Quantitative real‐time PCR (qPCR) can be used to quantify mRNA levels of senescence biomarkers; however, it faces challenges in correlating mRNA levels with protein expression. This discrepancy is particularly evident with p21 and p16, where mRNA levels often fail to reach significant thresholds needed for reliable identification of senescence.

Given the current limitations in detecting and analysing senescent cells, there is an urgent need for more robust methods. Existing senescence biomarkers lack specificity and reliability, and no consensus has been reached on their optimal detection. This study aimed to address these limitations by developing a robust method for analysing senescence markers using live single‐cell data and high‐content fluorescence microscopy. We assessed multiple senescence markers in both live and fixed cells across two distinct models of cellular senescence in human dermal fibroblasts.

## 2 Methods

### 2.1 Cell Culture

**FIGURE 1:**
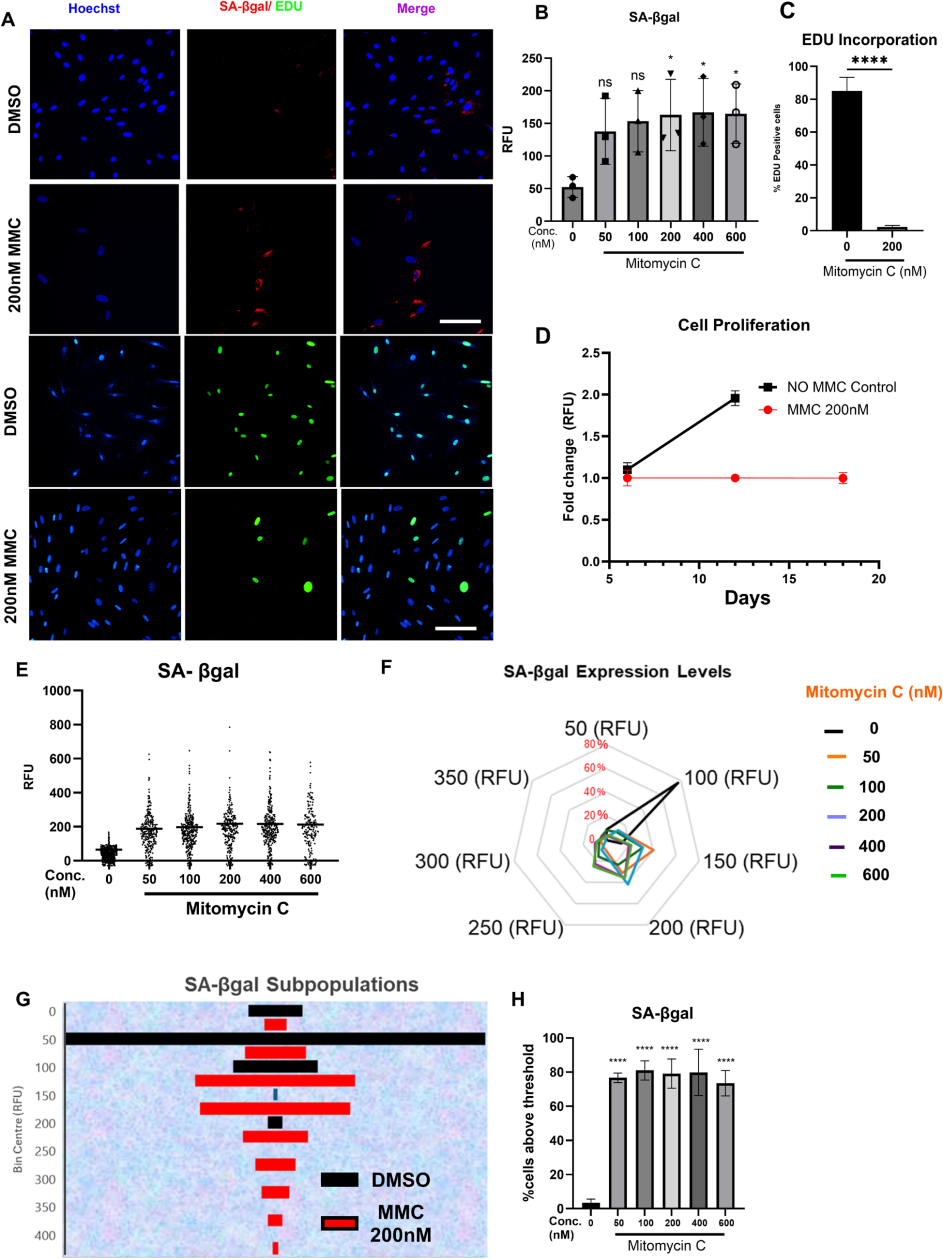
Assessment of SA‐βgal and cell proliferation in an MMC‐induced senescence model. (A) Representative images of SA‐βgal staining and EDU incorporation in HDFs treated with vehicle and 200 nM MMC. Images obtained with an IN‐Cell analyser 2200 (SA‐βgal) and Opera Phenix plus (EDU). Images show nuclei stained with Hoechst (blue), SA‐βgal (red)/EDU (Green) and merged image (scale bar = 100 μm). (B) Average fluorescence intensities of SA‐βgal in HDFs treated with vehicle (0.1% DMSO) or different concentrations (50–600 nM) of MMC; Error bars represent mean ± standard deviation from three independent biological replicates. *p < 0.05 (Simple one‐way ANOVA compared with the (0) control group). (C) Percentage of cells positive for EDU incorporation in HDFs treated with MMC; ****p < 0.0001, (Student unpaired *t*‐test compared with 0 group). (D) Time course effect of cellular proliferation on HDFs with MMC. (E) Single cell fluorescence intensities of SA‐βgal in HDFs treated with vehicle (0.1% DMSO) or different concentrations (50–600 nM) of MMC. (F) Radar chart of different SA‐βgal fluorescence intensities in HDFs treated with vehicle or different concentrations of MMC. (G) Sub‐population analysis of SA‐βgal fluorescence intensities in HDFs treated with vehicle or 200 nM MMC. (H) Percentage of cells with SA‐βgal intensity of greater than that of the threshold set in the control cells from the respective histograms. Error bars represent mean ± standard deviation from three independent biological replicates. ****p < 0.0001 (Simple one‐way ANOVA compared with the (0) control group).

**FIGURE 2:**
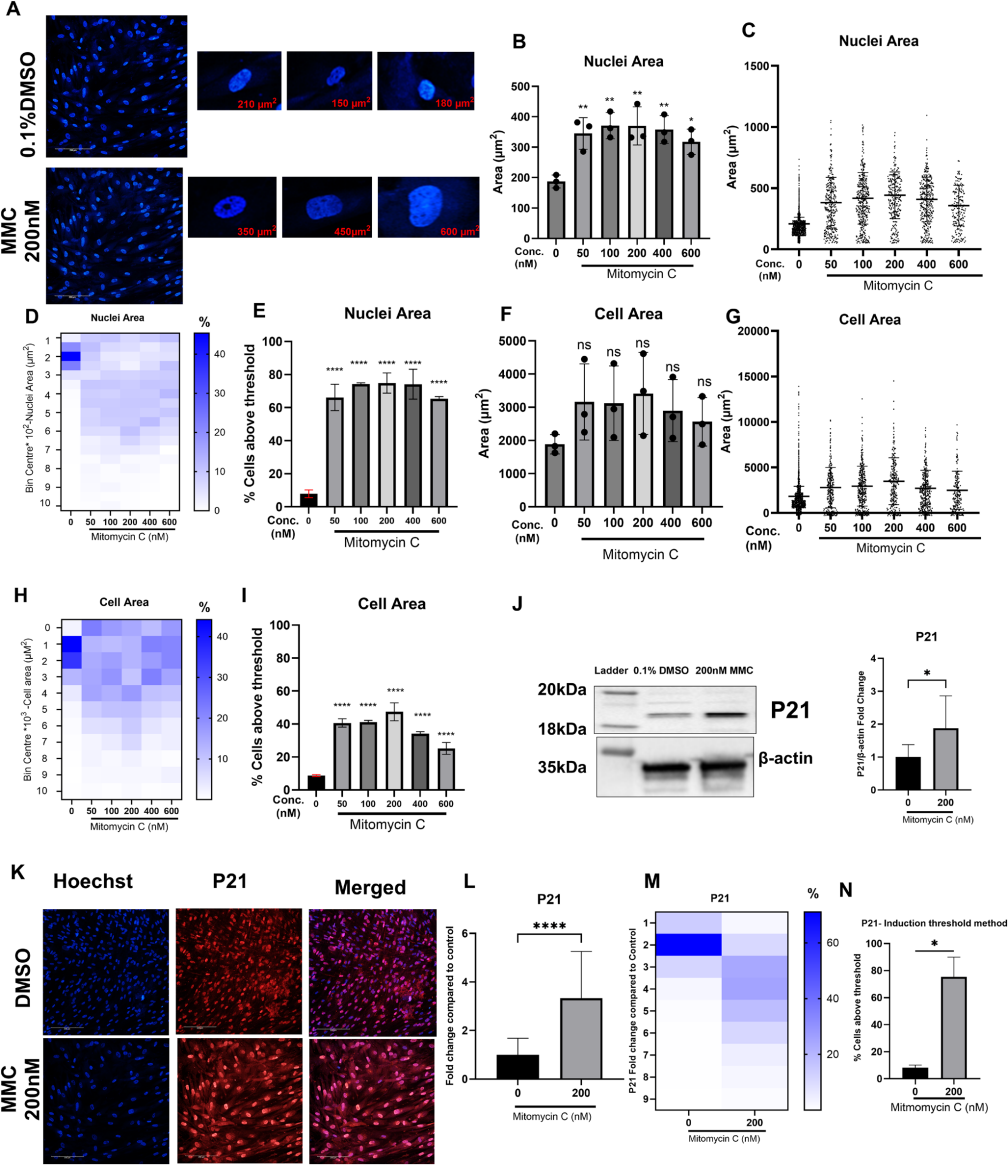
Assessment of nuclear area, cell area and P21 expression in an MMC‐induced senescence model in HDFs. Representative images were taken using Opera Phoenix plus at 20× magnification. (A) Representative image of Hoechst‐stained nuclei in HDFs treated with vehicle and 200 nM MMC treated HDFs. Average value of nuclei area (B), cell area (F), P21 (L) in vehicle and MMC treated HDFs; Error bars represent mean ± standard deviation from three independent biological replicates. For Nuclei and cell area (ns, not significant, *p < 0.05 **p < 0.01) (one‐way ANOVA compared with the (0) control group). For P21 (unpaired *t*‐test compared to the control (0) group ****p < 0.0001). Single‐cell data for nuclear area (C) and cell area (G) in HDFs treated with MMC. Individual cell‐derived histogram data categorised into various bin centres for nuclear area (D), cell area (H) and P21 (M) in vehicle‐ and MMC‐treated HDFs. (J) Representative image and quantitation of western blot showing the expression of P21 and β‐Actin, along with the relative quantitiation of expression of P21/β‐Actin expression in MMC‐treated HDFs; *p < 0.05, (Student's unpaired *t*‐test compared with 0 control group). (K) Representative images of P21 expression in the nuclei of HDFs treated with vehicle and 200 nM MMC, with nuclei labelled with Hoechst (blue), P21 (red) and merged image (scale bar: 10 μm). Percentage of cells with nuclear area (E), cell area (I) and P21 expression (N) exceeding the threshold set in the control cells, derived from the respective heatmaps in vehicle‐ and MMC‐treated HDFs. Error bars represent the mean ± standard deviation from three independent biological replicates. For nuclear area and cell area (****p < 0.0001, one‐way ANOVA compared to the control group); For p21 (****p < 0.0001, unpaired *t*‐test compared to the control group).

Primary human skin fibroblasts (HDFs) (106‐05 N, Sigma‐Aldrich, MO, USA, for Figures 1 and 2A–I; or PCS‐201‐010, ATCC, for the remaining figures) were cultured in Dulbecco's Modified Eagle Medium (DMEM, D5523, Sigma‐Aldrich) supplemented with 10% foetal bovine serum (FBS). Cells were maintained in a humidified incubator at 37°C with 5% CO_2_. Cells used for experiments were at passages 5–8 (ATCC) and less than 15 (Sigma‐Aldrich) for accelerated senescence models.

Renal proximal tubular epithelial cells (RPTEC) (CC‐2553, LONZA, Walkersville, USA) were cultured in renal basal epithelia media (CC‐3910, Lonza). Cells were maintained in a humified incubator at 37°C with 5% CO_2_. Cells used for experiments were from passage 1 to 3.

For chemotherapy‐induced senescence, cells were exposed to various concentrations (50–600 nM) of Mitomycin C (M4287, Sigma‐Aldrich) for 48 h, followed by a media change to non‐Mitomycin C media for an additional five days. For oxidative stress‐induced senescence, cells were treated with different concentrations (3.125–200 mM) of D‐galactose (D0750, Sigma‐Aldrich) for seven days.

For the induction of replicative senescence, HDFs were passaged every week and population doublings were calculated at each passage using the formula: population doubling = [(ln (Harvested cell number)−ln (seeded cell number))]/ln (2). Cells were considered senescent when the population doublings were consistently less than 0.2 across two consecutive passages. Early‐passage HDFs were defined as cells seeded at passage number less than 15, while late‐passage HDFs were defined as those at passage number greater than 30 with population doubling of less than 0.2 for two successive passages.

For rapamycin treatment, HDFs were pre‐incubated with 500 nM rapamycin for 24 h before the media was replaced with media containing 200 nM MMC + 500 nM rapamycin and incubated for an additional four days. Subsequently, the media was changed to media containing 500 nM rapamycin for a further three days, after which senescence biomarker assays were performed.

For assessment of the effect of rapamycin on replicating cells, replicating HDFs were treated with rapamycin 125–1000 nM for seven days, during which media was changed to rapamycin‐containing media on day four, after which the senescence biomarker assays were performed.

For assessment of concentration response of rapamycin on senescent cells, HDFs were plated in black 96 well plates (655090, μClear, Greiner). 24 h after plating, media was changed to MMC 200 nM containing media along with rapamycin 125–1000 nM. Four days post treatment, the media was changed to rapamycin 125–500 nM containing media for another three days, after which the senescence biomarker assessment was done.

### 2.2 Live Single‐Cell Assessment of SA‐β‐Gal

Cells were seeded at a density of 1.5 × 10^6^ in T‐25 flasks (430629, Corning). After 48 h, cells were treated with different concentrations of MMC (50, 100, 200, 400 and 600 nM) for 4 days, after which the media was changed to non‐MMC‐containing media. For D‐galactose‐induced senescence, cells were plated at a density of 500 cells per well in glucose‐containing media for 24 h, followed by a media change to glucose‐free media with the addition of D‐galactose at concentrations of 3.125, 6.25, 12.5, 25, 50, 100 and 200 mM. Seven days post‐induction, cells were seeded at a density of 500 cells per well in black 96‐well plates (655090, μClear, Greiner, Germany). After 24 h, SA‐β‐gal activity was assessed using the FastCellular Senescence SPiDER‐βGal Detection Kit (Product Number 092690301, MP Biomedicals, CA, USA).

Following the manufacturer's protocol, cells were washed with Hank's Balanced Salt Solution (HBSS) and incubated with Bafilomycin A1 solution for 1 h, followed by a 45‐min incubation with SPiDER‐βGal, Bafilomycin A1 and 5 μM Hoechst solution (62249, Thermo Fisher Scientific) to stain the nucleus. Cells were then washed twice with HBSS, and plates were prepared for imaging. Images were recorded using high‐content cell imaging systems, either the IN Cell Analyzer 2200 (GE Healthcare Life Sciences, IL, USA) at 10× magnification or the Opera Phenix Plus (Revvity, MA, USA) at 20× magnification, with excitation at 475 nm and emission at 594 nm. Images were analysed using IN Carta Image Analysis Software (GE Healthcare Life Sciences) or Harmony software, version 5.1 (Revvity).

Single‐cell mean fluorescence values and total fluorescence (Mean intensity × area of the mask) were quantified by the software. Sub‐population data analysis was conducted by binning fluorescence values at intervals specified in the figures, using GraphPad Prism version 9 and above. The threshold induction method was applied by setting a threshold value where at least 90% of values in vehicle‐treated cells (0.1% DMSO) fell, and the parameter was reported as the percentage of cells exceeding this threshold. The induction threshold was set at 90% of the values observed in vehicle‐treated cells, as 90% of these cells incorporated EdU, while the remaining 10% entered either a quiescent or senescent state. This induction threshold was also applied to the other biomarkers mentioned below.

### 2.3 Cell Synchronisation

HDFs were seeded into black, clear‐bottom 96‐well plates (655090, μClear, Greiner). After 24 h, the culture medium was replaced with serum‐free medium, and cells were incubated for 24 h to induce cell cycle synchronisation. Following synchronisation, the medium was replaced with serum‐containing medium containing either vehicle (0.1% DMSO) or mitomycin C (50–400 nM). The cells were incubated for 4 days. Post four days of MMC treatment, the medium was replaced with complete growth medium containing serum, and cells were cultured for an additional three days. Senescence biomarker analysis was performed at the end of this period.

### 2.4 Immunofluorescence Analysis

To assess P21^WAF1/CIP1^ expression, cells were seeded at a density of 500 cells per well in black 96‐well plates (655090, μClear, Greiner). Post seeding (48 h), cells were treated with 200 nM MMC. Seven days post‐senescence induction by MMC, cells were fixed with 4% formaldehyde for 10 min and permeabilized with 0.5% Triton X‐100 in phosphate‐buffered saline (PBS) for 10 min. Cells were then blocked with 10% normal goat serum (G9023; Sigma‐Aldrich) in PBST (0.1% Tween‐20 in PBS) for 1 h. After blocking, cells were incubated with primary antibodies (p21 WAF1/CIP1 Polyclonal Antibody, 14‐6715‐81, eBioscience, Invitrogen) at 1:50 dilution, p16INK4a Monoclonal Antibody (1D7D2, Invitrogen) at 1:100 dilution, Phospho‐Histone H2A.X (Ser139) Monoclonal Antibody (3F2) at 1:500 dilution overnight at 4°C. Secondary antibodies for p21 (2 μg/mL concentration, Alexa Fluor 647, Catalog #A‐21237, Thermo Fisher Scientific) and p16 (2 μg/mL concentration, Alexa Fluor 488, Catalog #A‐10631, Thermo Fisher Scientific) were added to the cells for 1 h at room temperature (RT). Nuclear staining was performed using Hoechst dye (5 μM). To obtain a proper cell mask, HDFs were incubated with HCS CellMask Stain (H32713, Invitrogen) at a dilution of 1:10,000 in PBST for 30 min and washed with PBS two times subsequently. Images were recorded using the Opera Phenix Plus (Revvity) at 20× magnification. Harmony 5.1 software was used to analyse the images.

### 2.5 EDU Incorporation

To assess EdU (5‐ethynyl 2′‐deoxyuridine) incorporation following MMC treatment, cells were treated with 50 and 200 nM MMC. After 48 h, the cells were cultured in non‐MMC‐containing media, and on day 5 post‐MMC treatment, the media was changed to EdU‐containing media (5 μM) for 48 h. After this incubation, cells were fixed with 4% paraformaldehyde (PFA) for 10 min and permeabilized with 0.1% Triton‐X100. EdU incorporation was then assessed using the Click‐iT Plus EdU Cell Proliferation Kit for Imaging with Alexa Fluor 488 dye (C10637, Thermo Fisher Scientific).

### 2.6 Western Blotting

10 μg of protein was combined with 5 μL of Laemmli buffer (1:9 β‐mercaptoethanol [PCS 1610710, Bio‐Rad Laboratories, Hercules, MA, USA] to Laemmli sample buffer [PCS 161‐0737, Bio‐Rad Laboratories]). It was combined with water sufficient to reach a final volume of 20 μL. Samples were heated at 95°C for 5 min, then loaded onto 4%–15% Mini‐PROTEAN TGX pre‐cast gels (PCS 4561084, Bio‐Rad Laboratories), and subsequently transferred onto PVDF membranes. Membranes were blocked for 1 h with 5% milk powder in Tris‐buffered saline with 0.1% Tween‐20 (TBST) and then washed before overnight incubation at 4°C with primary antibody diluted in 5% milk powder in TBST. The primary antibody used was rabbit anti‐P21 (PCS 14‐6715‐81, Thermo Fisher, MA, USA). After five washes with 5% TBST (5 min each), membranes were incubated for 1 h with secondary antibody goat anti‐rabbit IgG (PCS 1706515, Bio‐Rad Laboratories) at a 1:3000 dilution in 5% milk powder in TBST. After washing the membranes five additional times with 5% TBST (5 min each), they were incubated in Clarity Western ECL Blotting Substrate (PCS 1705060, Bio‐Rad) for 4 min. Blot images were captured using the ChemiDoc XRS+ imaging system (Bio‐Rad Laboratories), and densitometry was performed using Image Lab software. Following P21 imaging, membranes were washed five times with 5% TBST (5 min each) and incubated for 1 h at room temperature with mouse anti‐β‐actin primary antibody (PCS A5441, Sigma‐Aldrich, MA, USA) diluted 1:10000 in 5% milk powder in TBST. The membranes were then washed five times in 5% TBST (5 min each) and incubated with goat anti‐mouse IgG secondary antibody (PCS 1706516, Bio‐Rad) at a 1:3000 dilution in 5% milk powder in TBST for 1 h at room temperature. Following a final set of five washes in 5% TBST (5 min each), the membranes were incubated in Clarity Western ECL Blotting Substrate (PCS 1705060, Bio‐Rad) for 4 min. Blot images were captured using the ChemiDoc XRS+ imaging system (Bio‐Rad), and densitometric analysis was performed using Image Lab software.

### 2.7 Cell Proliferation Assay

HDFs treated with MMC, and control cells treated with vehicle (0.1% DMSO) were plated at a density of 10^4^ cells per well in black 96‐well plates (655090, μClear, Greiner). Cell proliferation was assessed by measuring fluorescence using the PrestoBlue Cell Viability Reagent (A13261, Thermo Fisher Scientific) on days 6, 12 and 18, according to the manufacturer's protocol.

### 2.8 Cell Death Assay

To assess cell death, HDFs were treated with MMC as described above and were stained for SA‐βgal enzyme activity at day 7. Post SA‐βgal staining, HDFs were incubated with 5 μM propidium iodide (P1304MP, Thermo Fisher Scientific) in HBSS for 15 min and then the HDFs were washed twice further with HBSS and imaged using Ex/Em 590/617 in IN Cell analyser 2000 (GE health sciences).

### 2.9 Capsase3/7 Activation Assay

To assess caspase3/7 activation, HDFs were seeded in T‐25 flasks, and senescence was induced in HDFs using MMC and D‐galactose as described above. Post senescence induction, HDFs were seeded in black 96‐well plates (655090, μClear, Greiner). Twenty‐four hours post seeding, HDFs were incubated with caspase3/7 reagent (R37111, Invitrogen) (2 drops for 1 mL) in DMEM low glucose without phenol red (11054020, Gibco) supplemented with 10% FBS along with ABT‐263 (79381S, New England Biolabs, Ipswich, MA, USA) for 1 h at 37°C incubator at 5 μM concentration. Post 1 h incubation, the HDFs were imaged using brightfield and Alexa 488 channel (Ex/Em: 488/510) using Opera Phenix Plus (Revvity) at 10× magnification every 30 min for 12 h.

### 2.10 Mitochondrial Superoxide Production

To measure mitochondrial superoxide production, HDFs were seeded in T‐25 flasks and senescence was induced in HDFs using MMC and D‐galactose as described above. Post senescence induction, HDFs were seeded in black 96‐well plates (655090, Greiner). 24 h post seeding, HDFs were incubated with MitoSOX Green (M36005, Thermo Fisher Scientific) at a concentration of 5 μM for 30 min. Fluorescence images (Ex/Em 488/510 nm) were obtained and quantified using Opera Phenix plus (Revvity).

### 2.11 Statistical Analysis

Statistical analyses were performed using GraphPad Prism (version 9.1 and higher, GraphPad Software, CA, USA). Data are reported as mean ± standard deviation (SD), with *p* values of < 0.05 considered statistically significant. Specific statistical tests and significance values for each experiment are provided in the corresponding figure legends.

## 3 Results

We first aimed to develop a chemotherapy‐induced accelerated model of senescence in HDFs using a live‐cell fluorescent assay. Our goal was to establish a robust method for senotherapeutic drug discovery based on a universally accepted hallmark of senescence, SA‐βgal. To achieve this, we assessed the enzymatic activity and fluorescence intensity of SA‐βgal, a key senescence marker, seven days after exposing cells to various concentrations of mitomycin C (MMC) using high‐content fluorescence microscopy.

We observed a concentration‐dependent increase in SA‐βgal enzymatic activity, with a statistically significant three‐fold increase beginning at 200 nM MMC treatment (Figure 1B). To assess the proliferation state of MMC‐treated HDFs, we examined EdU incorporation and found a 16‐fold decrease in the number of EdU‐positive cells compared to controls (Figure 1C). However, a small subpopulation, approximately 5% of MMC‐treated HDFs, still incorporated EdU into their nuclei. Given the presence of this small EdU‐positive subpopulation, we further investigated whether these cells exhibited enhanced proliferation over time. We conducted a time‐course assay to assess proliferation at multiple time points, revealing no increase in proliferation at 12 and 18 days post‐MMC treatment, while untreated cells exhibited a two‐fold increase in proliferation over the 12‐day period (control cells were assessed only until day 12, as they became confluent, leading to contact inhibition) (Figure 1D).

Since the SA‐βgal assay was performed using a high‐content microscope capable of generating single‐cell data, we plotted SA‐βgal fluorescence intensity levels of individual cells. This analysis revealed significant heterogeneity in SA‐βgal activity among MMC‐treated cells (Figure 1E). To further explore this heterogeneity, we performed a sub‐population analysis by binning the single‐cell fluorescence values of SA‐βgal enzymatic activity into specific bin centres (Figure 1F). We found that the SA‐βgal fluorescence values of vehicle‐treated HDFs clustered around 100 RFU, while most MMC‐treated HDFs (50–600 nM) clustered at 150 and 200 RFU. Further analysis of a single MMC concentration (200 nM) identified distinct sub‐populations with unique bin centres for SA‐βgal fluorescence intensities (Figure 1G). This heterogeneity explains why we observed only a three‐fold increase in mean fluorescence intensity in MMC‐treated groups compared to controls, as shown in Figure 1B.

To address this heterogeneity, we defined an ‘induction threshold’ for SA‐βgal activity based on the fluorescence intensity below which 90% of the control group's fluorescence values fell (detailed in the Methods section). Cells with fluorescence levels exceeding this threshold were classified as SA‐βgal positive. Our analysis showed an approximately 19‐fold increase in the percentage of SA‐βgal‐positive cells in all MMC‐treated groups (~80%) compared to controls (4.2%) (Figure 1H).

We next extended our investigation to other commonly used senescence biomarkers, including nuclear area, cell area and p21 expression. MMC treatment resulted in a significant 1.5‐ to 2‐fold increase in the average nuclear area in HDFs (Figure 2A,B). Similar to the SA‐βgal data, considerable variation in nuclear area was observed among individual cells (Figure 2C). Sub‐populations with distinct nuclear areas were evident, as confirmed by differential clustering in the corresponding heatmap (Figure 2D). Applying our induction threshold method to nuclear area across three independent trials revealed a consistent and robust 6‐ to 7‐fold increase in MMC‐treated groups compared to vehicle‐treated controls (Figure 2E).

We also assessed cell area, a cytoskeletal biomarker of senescence. Our results showed a 1.8‐fold increase in cell area in HDFs treated with50–200 nM MMC, though cell area decreases at higher MMC concentrations (Figure 2F). These increases in cell area were not statistically significant. Nonetheless, single‐cell data and heatmap analysis displayed trends similar to those observed for SA‐βgal and nuclear area (Figure 2G). The percent frequency distribution of cell areas revealed the emergence of larger cell populations in MMC‐treated groups compared to the control (Figure 2H). Further analysis using the induction threshold method showed a statistically significant 4.5‐fold increase in cell area in cells treated with 200 nM MMC compared to the control group (Figure 2I).

Another key biomarker for identifying senescent cells both in vitro and in vivo is p21, a downstream target of TP53 that plays a central role in cell cycle arrest and has various other physiological functions. Initially, we measured P21 expression conventionally using western blot analysis and observed a statistically significant two‐fold increase in HDFs treated with 200 nM MMC (Figure 2J).

Next, we assessed p21 expression at a single‐cell level using high‐content microscopy (Figure 2K), which revealed a statistically significant three‐fold increase in p21 levels in MMC‐treated HDFs. However, further single‐cell analysis uncovered substantial heterogeneity in p21 expression, as illustrated by the scattered histogram (Figure 2M). Applying the induction threshold method showed a 7.5‐fold increase in p21 expression in MMC‐treated HDFs (Figure 2N), providing a more robust and accurate assessment of this senescence biomarker compared to the traditional method of averaging values.

To assess the time‐dependent expression of senescence markers, we performed a time‐course analysis of these senescence biomarkers along with p16, another key hallmark marker of senescence (Figure S1). Analysis at 24, 48, 72 and 96 h revealed a progressive increase in the levels of all markers over time, with peak expression observed at 96 h.

In addition to MMC, we used D‐galactose to induce accelerated senescence in HDFs. The concentration‐dependent effects of D‐galactose on senescence markers, along with similarly heterogeneous expression patterns, are presented in Figures S2 and S3.

To confirm the validity and robustness of MMC and D‐galactose as senescence inducers, we assessed oxidative stress and DNA damage in HDFs following treatment. Both agents significantly increased mitochondrial superoxide production (Figure S4A,B). DNA damage, assessed via γH2AX staining following MMC treatment, was evident from as early as 24 h (Figure S4C,D), confirming its genotoxic effect. These findings support the efficacy of both MMC and D‐galactose in inducing senescence in HDFs. Additionally, propidium iodide (PI) staining was performed to rule out cell death in HDFs treated with sub‐toxic concentrations of MMC. No considerable increase in PI‐positive cells was observed up to 200 nM MMC concentration, confirming the absence of cell death under these conditions (Figure S5).

**FIGURE 3:**
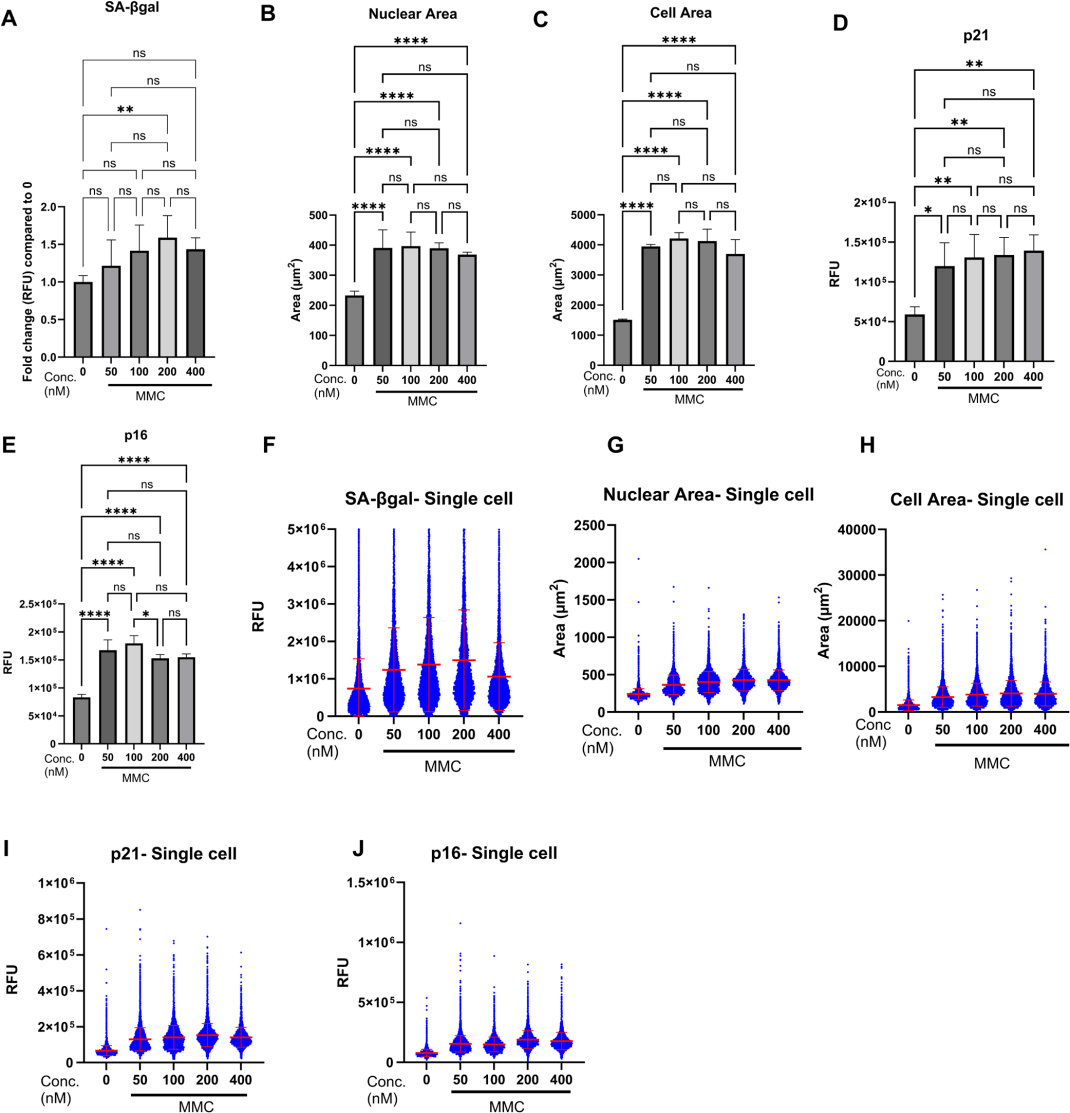
Assessment of senescence biomarkers in MMC‐induced senescent HDFs with synchronised cell cycles. Average total fluorescence intensities of SA‐βgal (A), nuclear area (B), cell area (C), nuclear p21 (D) and nuclear p16 (E) in HDFs with synchronised cell cycle treated with vehicle (0.1% DMSO) or different concentrations (50–400 nM) of MMC; Error bars represent mean ± standard deviation from three independent biological replicates. Ns: Not significant, *p < 0.05, **p < 0.01, ***p < 0.001, ****p < 0.0001 (One‐way ANOVA with Dunnet's post hoc analysis; Mean of all group compared with each other). Single cell fluorescence intensities of SA‐βgal (F), nuclear p21 (I) and nuclear p16 (J) in HDFs treated with vehicle (0.1% DMSO) or different concentrations (50–400 nM) of MMC. Single cell data of nuclear area (G) and cell area (H) in HDFs treated with vehicle (0.1% DMSO) or different concentrations (50–400 nM) of MMC.

To determine whether the heterogeneity observed in senescence marker expression was attributable to differences in cell cycle phase, we evaluated senescence biomarkers in HDFs following cell cycle synchronisation. MMC treatment induced robust senescence in synchronised HDFs, as evidenced by a 1.5‐fold increase in SA‐β‐galactosidase activity after treatment with 200 nM MMC (Figure 3A), a mean 1.8‐fold increase in nuclear area (Figure 3B), and a threefold increase in total cell area (Figure 3C). In addition, the expression of the cell cycle inhibitors p21 and p16 increased by an average of 2.2‐fold and 3‐fold, respectively, across all tested MMC concentrations (Figure 3D,E).

Notably, single‐cell analysis revealed considerable heterogeneity in the expression of all examined biomarkers, including SA‐β‐gal activity (Figure 3F), nuclear area (Figure 3G), cell area (Figure 3H), p21 (Figure 3I) and p16 (Figure 3J). These findings indicate that the heterogeneity in senescence marker expression is not solely due to differences in cell cycle phase but rather reflects a genuinely heterogeneous senescence response following sub‐toxic chemotherapy exposure.

We also examined the replicative senescence model and observed a robust induction of senescence, as indicated by increased SA‐β‐gal activity, nuclear area and cell area (Figure S6). Consistent with the accelerated models, single‐cell analysis confirmed heterogeneous expression of senescence biomarkers, indicating the presence of a heterogeneous HDF population. These results indicate that cellular heterogeneity is a consistent feature of both replicative and stress‐induced senescence in HDFs.

**FIGURE 4:**
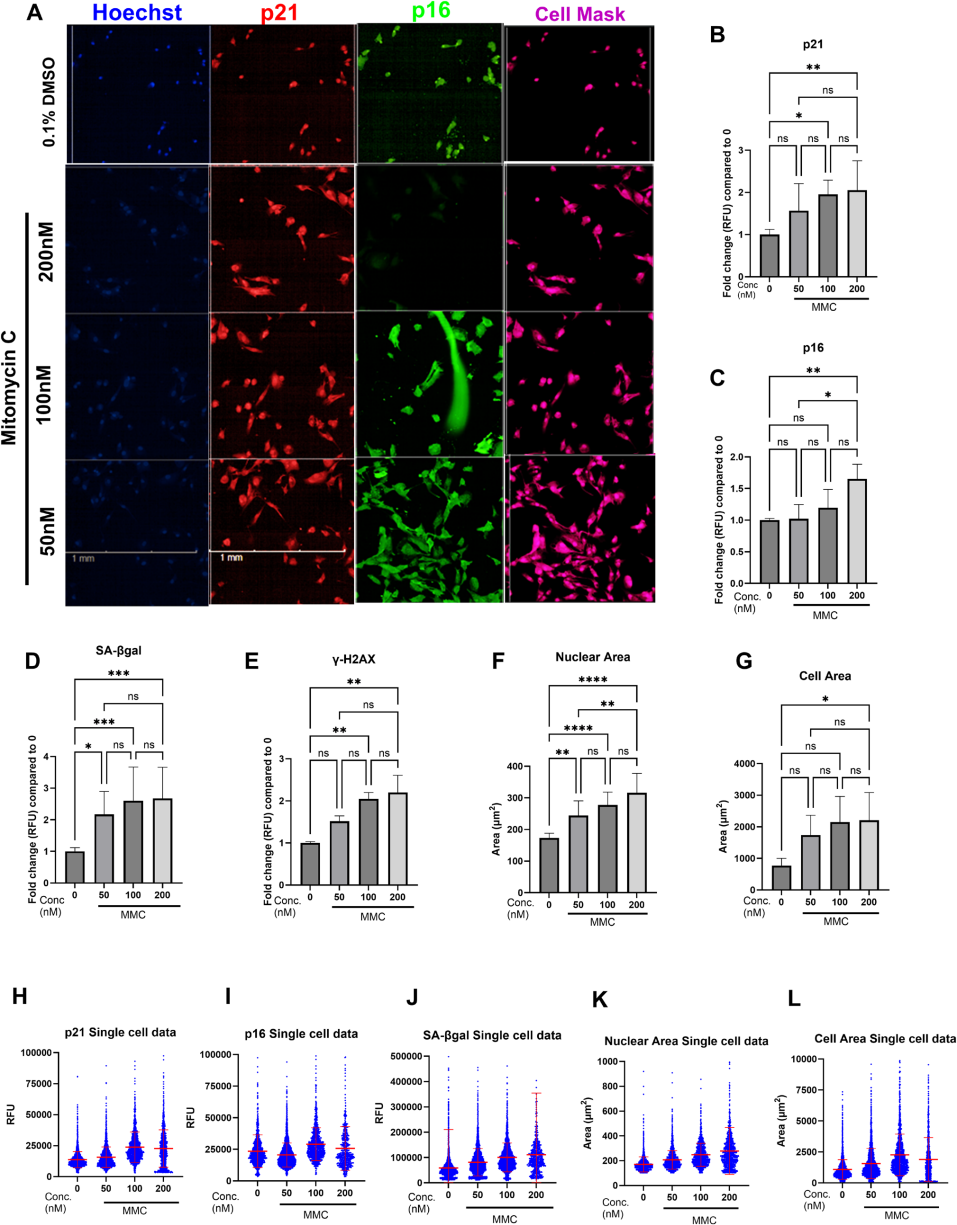
Assessment of senescence biomarkers in renal proximal tubule epithelial cells (RPTEC). Representative images were taken using Opera Phoenix plus at 10× magnification. (A) Representative image of Hoechst (Blue), p21 (Red), p16 (Green) and cell mask (Pink) in RPTECs treated with vehicle (0.1% DMSO) and 50–200 nM MMC: Scale bar—2 mm. Average fold change of total fluorescence of nuclear p21 (B) and p16 (C), SA‐βgal (D) and γ‐H2AX (E) along with nuclear area (F), cell area (G) in RPTEC's treated with vehicle and 50–200 nM MMC. Data are presented as mean ± SD from three biological replicates; Statistical analysis was performed using one‐way ANOVA followed by Tukey's post hoc test for multiple comparisons between all groups.; (ns, not significant; *p < 0.05, **p < 0.01, ***p < 0.001, ****p < 0.0001). Single cell data of nuclear p21 (H) and p16 (I), SA‐β‐gal (J) along with nuclear area (K) and cell area (L) in RPTEC's treated with vehicle and 50–200 nM MMC.

To determine whether MMC‐induced senescence and associated biomarker heterogeneity were specific to human dermal fibroblasts (HDFs) or also observed in other cell types, we assessed the effects of MMC in renal proximal tubular epithelial cells (RPTECs), a non‐fibroblast lineage relevant to aging and kidney pathology (Figure 4A). Treatment with 100 and 200 nM MMC induced a robust senescence response in RPTECs, as indicated by a 1.8‐fold increase in nuclear p21 expression (Figure 4B). A 1.5‐fold increase in nuclear p16 expression was also observed following treatment with 200 nM MMC (Figure 4C). In addition, SA‐β‐gal activity increased twofold (Figure 4D), and a similar twofold increase was seen in nuclear γH2AX levels (Figure 4E), indicating activation of the DNA damage response. Morphological changes associated with senescence were also evident, with nuclear area increasing by 1.8‐fold (Figure 4F) and cell area by approximately threefold (Figure 4G) in MMC‐treated RPTECs. Importantly, consistent with our observations in HDFs, single‐cell analysis revealed substantial heterogeneity in the expression of senescence biomarkers, including p21 (Figure 4H), p16 (Figure 4I), SA‐β‐gal activity (Figure 4J), nuclear area (Figure 4K) and cell area (Figure 4L). These findings suggest that both the induction of senescence and the heterogeneity in biomarker expression are conserved features across different cell types following MMC treatment.

**FIGURE 5:**
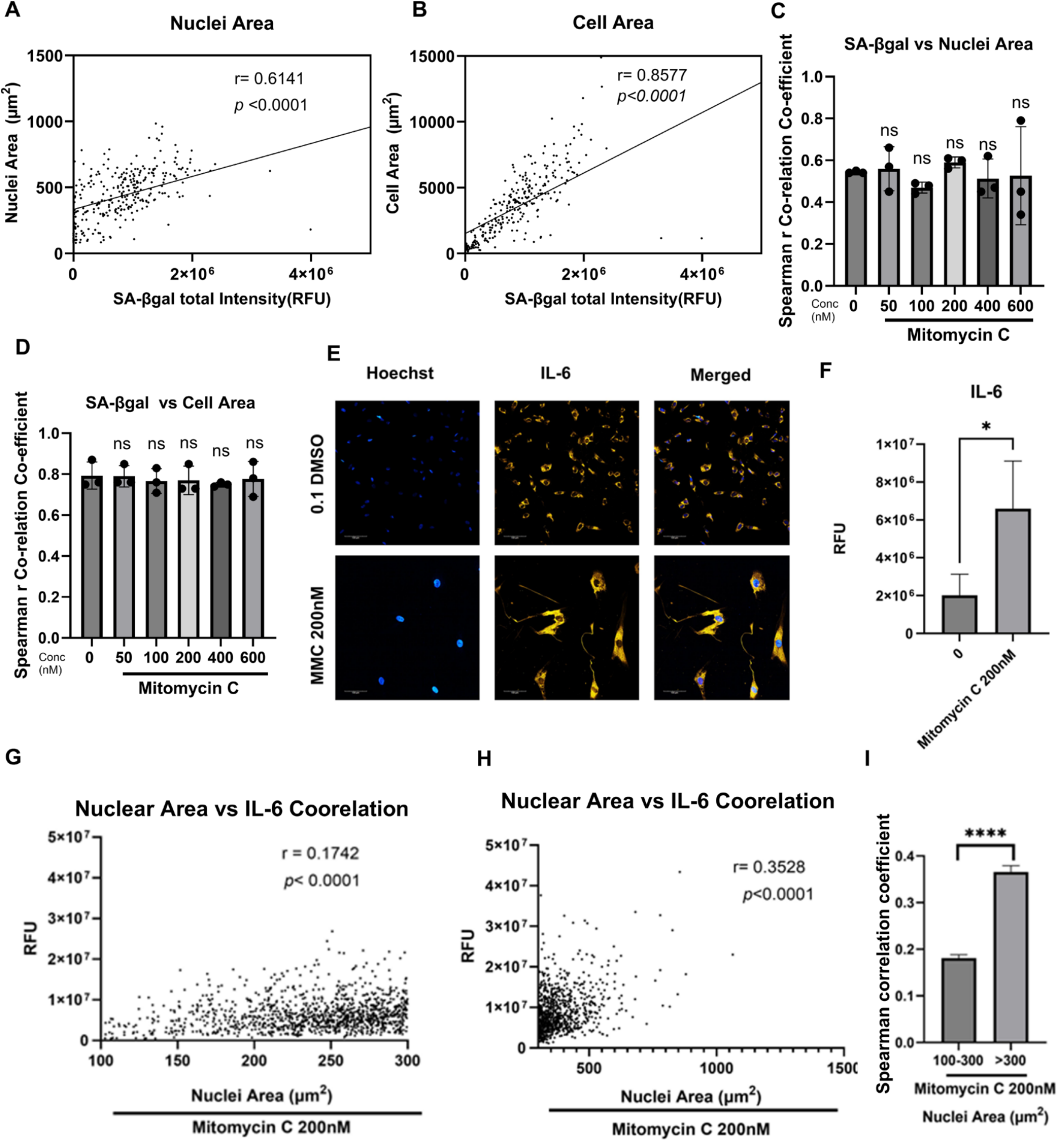
Correlation analysis of senescence‐associated biomarkers and the impact of cellular heterogeneity on the senescence‐associated secretory phenotype (SASP) in MMC‐induced senescence. Representative correlation plot of SA‐βgal total fluorescence intensity versus nuclear area (A) and ell area (B) for the MMC 200 nM group. Spearman correlation coefficient values for SA‐βgal total fluorescence intensity versus nuclear area (C) and cell area (D). Data represent mean ± standard deviation from three independent biological replicates; ns, not significant (*p >* 0.05, ordinary one‐way ANOVA with Dunnett's post hoc test compared to the control group). (E) Representative images of IL‐6 expression in HDFs treated with vehicle and 200 nM MMC with nuclei labelled with Hoechst (blue) and IL‐6 labelled (yellow); merged image (Scale bar: 10 μm). Images were captured using the Opera Phoenix plus at 20× magnification. (F) Average IL‐6 expression in HDFs treated with vehicle or 200 nM MMC. Representative correlation graph of nuclear area (100–300 μm^2^) versus IL‐6 (RFU) (G) and nuclear area (> 300 μm^2^) versus IL‐6 (RFU) (H). (I) Average Spearman correlation coefficient (*r*) of nuclear area subpopulations versus IL‐6 fluorescence in HDFs treated with 200 nM MMC. Error bars represent the mean ± standard deviation from three independent biological replicates (unpaired *t*‐test, *p < 0.05, ****p < 0.001).

To investigate the correlation between different senescent markers and determine if these markers can be used interchangeably, we generated correlation plots between SA‐βgal, nuclear area and cell area (Figure 5A,B). Positive correlations with r values of around 0.6 for nuclear area (Figure 5C) and 0.8 for cell area (Figure 5D) were observed. Notably, these r values were not statistically different between the control and treatment groups, suggesting the presence of heterogeneous populations across all groups.

A key characteristic of senescent cells is the acquisition of a secretory phenotype, known as the senescence‐associated secretory phenotype (SASP), which includes various cytokines, chemokines and metalloproteinases. Among these, IL‐6 is a major cytokine that has been extensively studied and consistently found to be elevated in senescent cells. We investigated IL‐6 expression in our accelerated senescence model (Figure 5E) and observed a three‐fold increase in IL‐6 expression in cells treated with 200 nM MMC (Figure 5F). However, significant heterogeneity in IL‐6 expression was evident, as indicated by the large error bars in Figure 5F.

To further explore this heterogeneity, we examined the correlation between nuclear area (100–300 μm^2^) (Figure 5G) and > 300 μm^2^ (Figure 5H) with IL‐6 expression levels. This analysis was performed based on our previous finding (Figure 2B), which showed that the nuclear area in vehicle‐treated HDFs was < 300 μm^2^ (Figure 5I). Our analysis revealed a statistically significant increase in the Spearman correlation coefficient for cells with larger nuclei (> 300 μm^2^) compared to those with nuclear areas between 100 and 300 μm^2^. This suggests that the expression of senescence biomarkers is associated with the pro‐inflammatory secretory phenotype of the cells.

**FIGURE 6:**
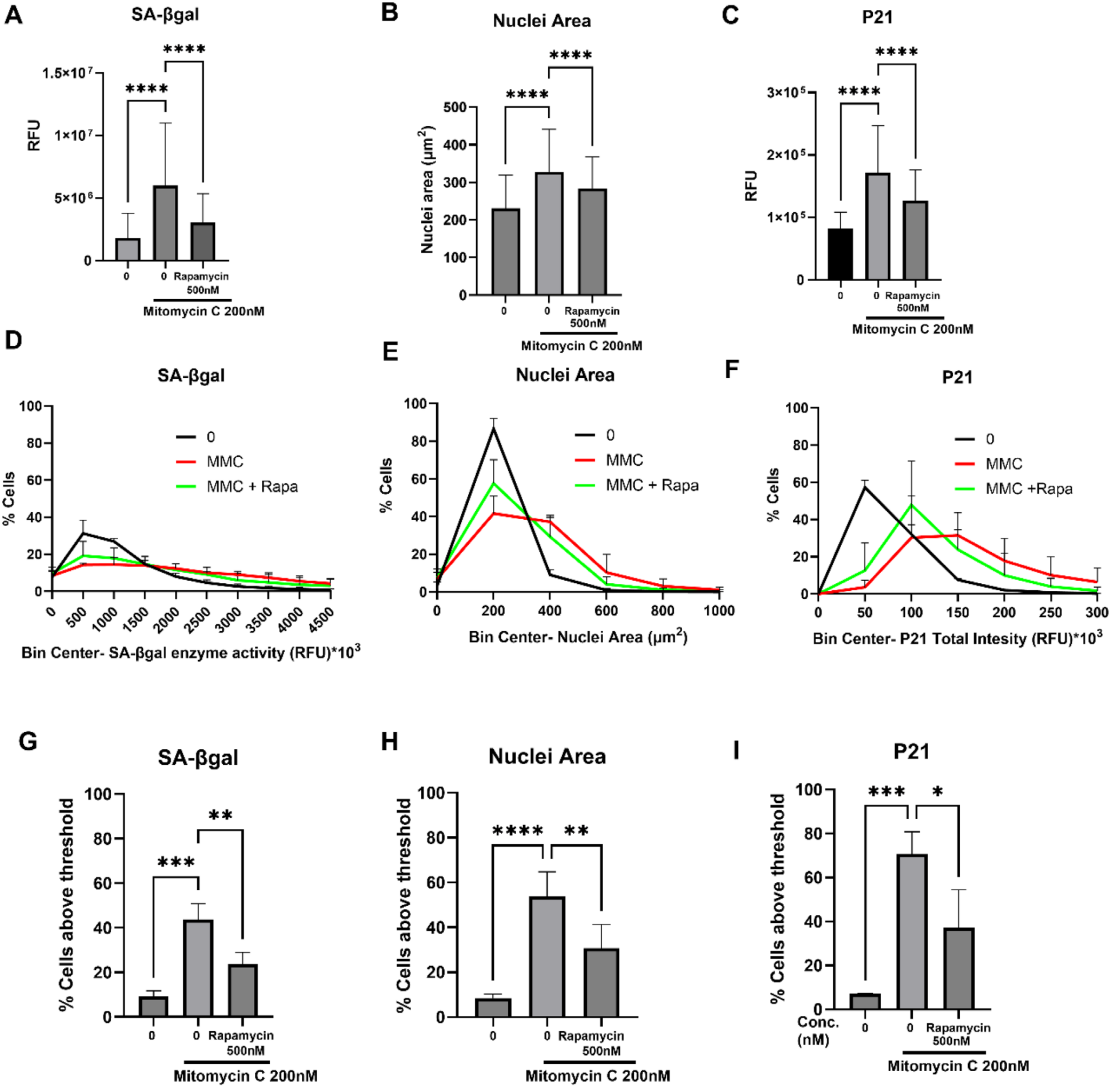
Assessment of senescence biomarkers in the MMC‐induced senescence model with rapamycin treatment. Average total fluorescence intensity of SA‐βgal (A), nuclear area (B) and P21 (C) in HDFs treated with either MMC or MMC plus rapamycin. Data represent the mean ± SD from *n* = 3 biological replicates; ***p < 0.001, ****p < 0.0001 (ordinary one‐way ANOVA compared to the MMC 200 nM treated group). Sub‐population analysis of SA‐βgal (D), nuclear area (E) and p21 (F) in HDFs treated with either MMC or MMC plus rapamycin. Percentage of cells having increased expression of SA‐βgal (G), nuclear area (H) and p21 (I) in HDFs treated either with MMC or MMC plus rapamycin using the induction threshold method. Data represents the mean ± SD from *n* = 3 biological replicates; *p < 0.05, **p < 0.01, ***p < 0.001, ****p < 0.0001 (ordinary one‐way ANOVA compared to the MMC 200 nM treated group).

To further investigate the heterogeneity of senescence biomarkers, we examined the effects of rapamycin, a well‐known senomorphic (Sturmlechner et al. 2021; Wang et al. 2017), on these markers. To determine the concentration of rapamycin with a senomorphic effect, we performed a concentration–response experiment using doses up to 1 μM to assess its ability to attenuate senescence biomarkers (Figure S7). Rapamycin exhibited peak efficacy in reducing SA‐β‐gal activity at 500 nM, and this concentration was therefore selected for subsequent experiments. Rapamycin treatment significantly decreased the single‐cell mean levels of SA‐βgal fluorescence (Figure 6A), nuclear area (Figure 6B) and nuclear p21 fluorescence (Figure 6C). Sub‐population analysis of these biomarkers revealed a rightward shift in values with MMC treatment compared to the control, while rapamycin reduced this rightward shift (Figure 6D–F). Applying the induction threshold method showed a statistically significant reduction in senescence biomarkers in the rapamycin‐treated group (Figure 6G–I).

To determine if the effects of rapamycin are specific to MMC‐induced senescence, we assessed senescence biomarkers in a D‐galactose‐induced model of senescence that we had established (Figures S4, S5). We observed a trend toward reduced nuclear p21 fluorescence intensity and a significant decrease in average nuclear and cell areas (Figure S8A–C). Sub‐population analysis revealed a rightward shift in the distribution curves of senescence biomarkers in HDFs cultured in D‐galactose media compared to those cultured in glucose media. For nuclear p21 expression, rapamycin slightly shifted the curve leftward (Figure S8D). Interestingly, for nuclear and cell areas, rapamycin‐treated HDFs in D‐galactose media showed a shift in the curve back to control levels observed in HDFs cultured in glucose media (Figure S8E,F). Using the induction threshold method, we observed a two‐fold reduction in nuclear P21 expression in rapamycin‐treated cells compared to D‐galactose‐only treated cells, while nuclear and cell areas exhibited a three‐fold reduction relative to the D‐galactose group (Figure S8G–I). To assess whether the rapamycin‐induced decrease in senescence markers is specific to senescent cells or also occurs in proliferating cells, we tested rapamycin in normally replicating cells. A similar reduction in senescence markers was observed, indicating that this effect is not specific to senescent cells (Figure S9). This suggests that rapamycin may suppress basal expression of these markers independent of a senescent state, warranting caution when interpreting its senomorphic activity.

Given the heterogeneous expression of senescence biomarkers, we next investigated whether specific subpopulations of senescent HDFs were more susceptible to senolytic treatment. Replicating or senescent HDFs, induced by either MMC or D‐galactose, were treated with the known senolytic agent ABT‐263, and Caspase 3/7 activation was assessed at multiple timepoints from 30 min to 12.5 h (Figure S10). While ABT‐263 had minimal effect on replicating cells, it induced robust Caspase 3/7 activation in nearly all senescent cell groups by the end of the time course, suggesting that its senolytic activity is not limited by the heterogeneous expression of senescence biomarkers.

## 4 Discussion

This study explored the heterogeneous expression of senescence biomarkers using single‐cell fluorescence microscopy, introducing a novel approach to analyse and quantify these biomarkers to account for diverse cell populations within a primary cell culture. At present, no single, universally accepted marker of senescence is available. Traditionally, SA‐βgal is assessed in fixed cells through histochemical analysis, which does not allow for quantitative studies of expression levels in senescent cells (Dimri et al. 1995). Fibroblasts, one of the gold‐standard models for studying cellular senescence, are not homogeneous in their replicative state, even at early passages. With each passage, a subset of cells progresses toward cellular senescence, leading to a heterogeneous population at different stages of the senescence program. This heterogeneity presents challenges in robustly assessing the efficacy of senotherapeutics for drug screening.

In this study, live‐cell imaging was used to reliably assess three senescence biomarkers including increased SA‐βgal activity and two morphological features, nuclear area and cell area, in live cells. SA‐βgal activity and nuclear area, in addition to increasing with D‐galactose and MMC treatment, displayed notable heterogeneity at the single‐cell level, revealing distinct sub‐populations within treated cells. Our findings indicate that in both chemotherapy‐induced and D‐galactose models of senescence, rapamycin treatment selectively impacted specific sub‐populations of senescence biomarkers. This highlights the importance of assessing heterogeneity within these biomarkers to better understand senescence dynamics. The physiological significance of these sub‐populations warrants further investigation.

The current data indicate a strong correlation between nuclear area and cell area with SA‐βgal activity. This suggests that a simple assessment of nuclear or cell area can be used interchangeably to determine senescence induction. One major disadvantage of SA‐βgal is its overexpression in confluent cells, which can lead to false‐positive results. This drawback can be addressed by assessing nuclear size, which is not affected by the confluency of fibroblasts.

Our data also reveal heterogeneity in the increase in nuclear area. We hypothesise that this may be due to a correlation between nuclear size and the SASP level, as evidenced by the stronger correlation of IL‐6 fluorescence levels with increasing nuclear area. However, further studies are required to confirm this association.

Our data show varying levels of nuclear p21 expression in senescent cells. Recent studies have revealed that p21 is involved in cell fate decisions and induces an independent biosecretome (Sturmlechner et al. 2021). Thus, these observed differences in P21 levels may suggest that, depending on p21 expression levels, cells may have distinct fates or an altered regulation of SASP secretion. Interestingly, a small population of MMC‐treated fibroblasts incorporated EdU into their nuclei, although these cells did not replicate over time. We hypothesize that this may be due to either paracrine effects from neighbouring cells or these cells' limited ability to repair and synthesise DNA, though not enough to proliferate into daughter cell populations.

In addition, p21 and TP53 expression is involved in mediating apoptosis (Yosef et al. 2017). Higher levels of p21 render cells more resistant to apoptosis, as senescent cells are known to exhibit anti‐apoptotic properties (Yosef et al. 2017). Therefore, drugs that reduce p21 levels might sensitise cells to senolytic therapies targeting the p53‐p21 axis in senescence.

To determine whether the observed heterogeneity was simply a consequence of differences in cell cycle phase, we repeated our senescence induction experiments in synchronised HDFs. Despite achieving synchronisation prior to MMC treatment, substantial heterogeneity in senescence biomarker expression was still evident at the single‐cell level. These findings indicate that the heterogeneity is unlikely to be solely due to cell cycle variation and instead reflects intrinsic variability in the cellular response to senescence‐inducing stimuli.

The induction threshold method described in this paper demonstrates that, due to varying expression levels of senescence biomarkers, analysing the entire population as a whole and averaging the data may not be the most effective approach for assessing senescence biomarkers. Indeed, we show that western blotting revealed approximately a two‐fold increase in p21 expression, similar to the average values observed using the fluorescence microscopy‐based method. However, the induction threshold method indicated a robust six to seven‐fold change, suggesting that averaging data might mask the efficacy of senotherapeutic compounds. Thus, this new method provides a more robust way to identify novel senotherapeutic drugs.

In addition to these advantages, the present study demonstrated that the induction threshold method can be applied to different senescence inducers, acting through various mechanisms, such as D‐galactose‐induced senescence. This is a key point of differentiation, as many senotherapeutic effects are limited to specific senescence effectors and cell types.

To validate the generalisability of our findings, we extended the senescence model to RPTECs, a non‐fibroblast epithelial lineage relevant to kidney ageing. Similar to HDFs, MMC‐treated RPTECs exhibited robust senescence induction and biomarker heterogeneity. These results confirm that the observed heterogeneity and applicability of the induction threshold method are not limited to fibroblasts but are also conserved across other physiologically relevant cell types.

The present study has demonstrated that the induction threshold method can be applied to senotherapeutic drug discovery, as evidenced by the senotherapeutic effect of rapamycin, a drug that is currently in phase 3 clinical trials for longevity extension and known as a senomorphic (Sturmlechner et al. 2021). We show that rapamycin significantly decreased all the aforementioned biomarkers of senescence. The main advantage of this method is that, by analysing biomarkers at the single‐cell level, the pharmacological effect size of the drug can be robustly demonstrated in vitro. Thus, this single‐cell analysis of senescence biomarkers is suitable for medium‐ to high‐throughput drug discovery screens.

Furthermore, the study evaluated the senolytic activity of ABT‐263, a BCL‐2 family inhibitor and demonstrated its ability to robustly induce caspase 3/7 activation in senescent cells, irrespective of biomarker heterogeneity. This suggests that while senescence markers are variably expressed, senolytic responses to ABT‐263 are not strictly dependent on specific biomarker expression levels. Such findings support the use of ABT‐263 in eliminating diverse subpopulations of senescent cells and highlight its value in preclinical senolytic screening.

In conclusion, this study highlights the heterogeneous expression of senescence biomarkers within a cell population using fluorescence microscopy and presents an alternative single‐cell method for the robust discovery of senotherapeutic drug candidates.

## Author Contributions

V.S. and I.A. conceived the study and designed the experiments. V.S., J.T., C.C., C.A., K.B. and Y.W. performed the experiments. V.S. and I.A. wrote the manuscript. I.A., N.G. and S.R. supervised the project. All authors reviewed the manuscript and provided intellectual input.

## Conflicts of Interest

The authors declare no conflicts of interest.

## Supporting information


**Figure S1:** acel70209‐sup‐0001‐FigureS1.docx.

## Data Availability

The data that support the findings of this study are available on request from the corresponding author.
