# Supplementary material for: Single‐Cell Fluorescence Imaging Reveals Heterogeneity in Senescence Biomarkers and Identifies Rapamycin‐Responsive Sub‐Populations
Source: Aging Cell. 2025 Sep 3;24(10):e70209. doi: 10.1111/acel.70209 (PMC12507413; doi:10.1111/acel.70209)
Supplement: Supplementary file 1 — Figure S1: acel70209‐sup‐0001‐FigureS1.docx. [file ACEL-24-e70209-s001.docx]

**Supplementary Figures**

**Single-cell fluorescence imaging reveals heterogeneity in senescence biomarkers and identifies rapamycin-responsive sub-populations**

Vijayraghavan Seshadri ^1,2^, Charmaine Chng ^1^, Joel Tyler ^1^, Cesta Adikerta ^1^, Kaveh Baghaei ^1^, Yan Wang^1^, Nuri Gueven ^2^, Sharon Ricardo ^1^, Iman Azimi ^1,2^*

^1^ Monash Biomedicine Discovery Institute, Department of Pharmacology, Monash University, Clayton 3168, Victoria, Australia.

^2^ School of Pharmacy and Pharmacology, College of Health and Medicine, University of Tasmania, Hobart, Tasmania, Australia

***** Correspondence: Iman Azimi, Monash University [iman.azimi@monash.edu](mailto:iman.azimi@monash.edu)


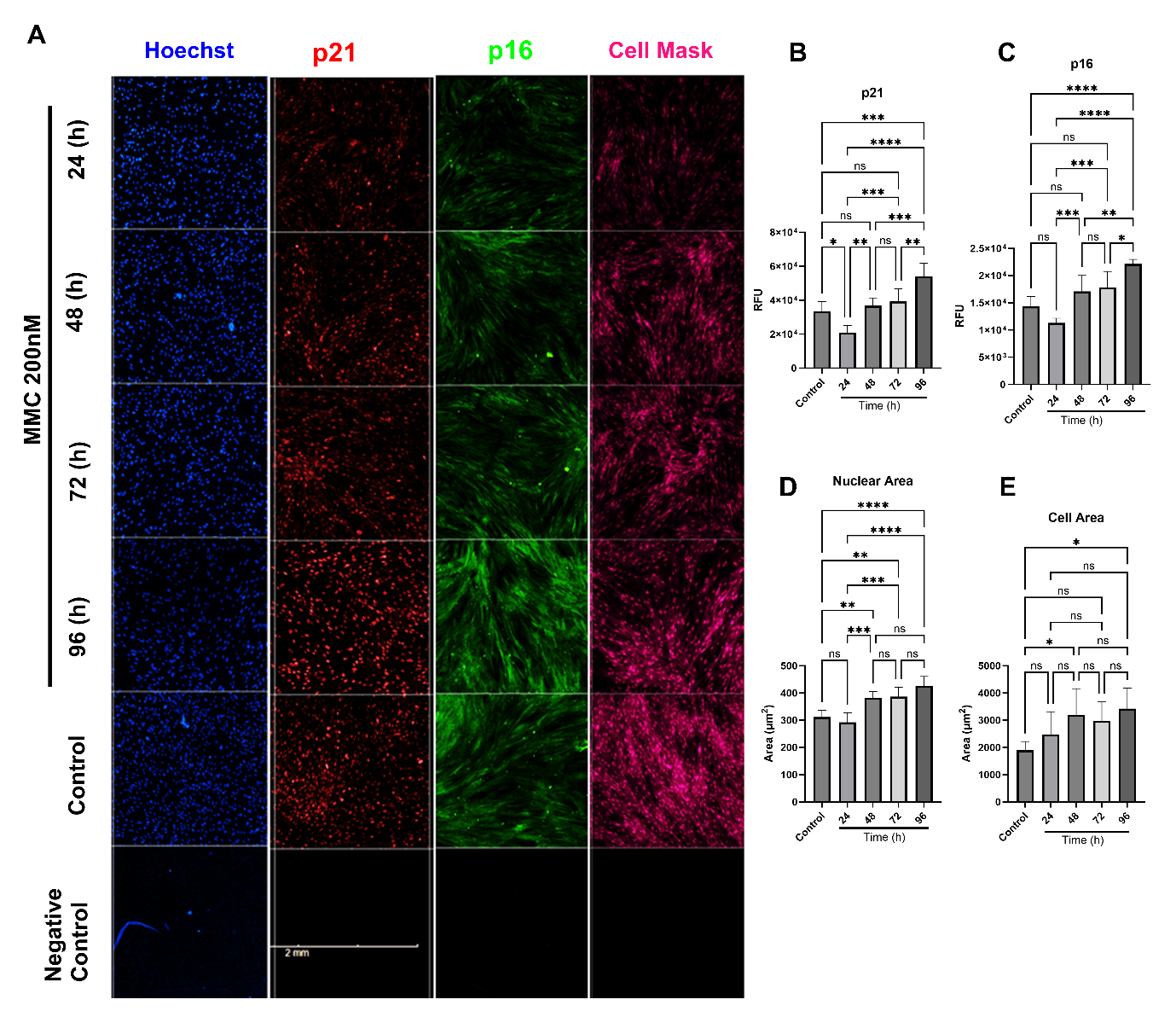


**Supplementary Figure 1. Time-course analysis of senescence biomarker expression in an accelerated senescence model**. Representative images were taken using Opera Phoenix plus ^TM^ at 10X magnification. 0 represents vehicle (0.1% DMSO) treated HDFs. **(A)** Representative image of Hoechst (Blue), p21 (Red), p16 (Green) and cell mask (Pink) in HDFs treated with 200 nM MMC at 24,48,72 and 96 hours; Scale bar: 1mm. Total fluorescence of nuclear p21 **(B)** and p16 **(C)** at 24,48,72 and 96 hours in HDFs treated with MMC 200nM. Total nuclear area **(D)** and cell area **(E)** at 24,48,72 and 96 hours in HDFs treated with MMC 200nM. Data are presented as mean ± SD from three biological replicates; Statistical analysis was performed using one-way ANOVA followed by Tukey’s post hoc test for multiple comparisons between all groups.; (ns- not significant, * *p* < 0.05, ** *p* < 0.01, *** *p*< 0.001, **** *p* < 0.0001). Wells with “Negative Control” did not receive secondary antibody.


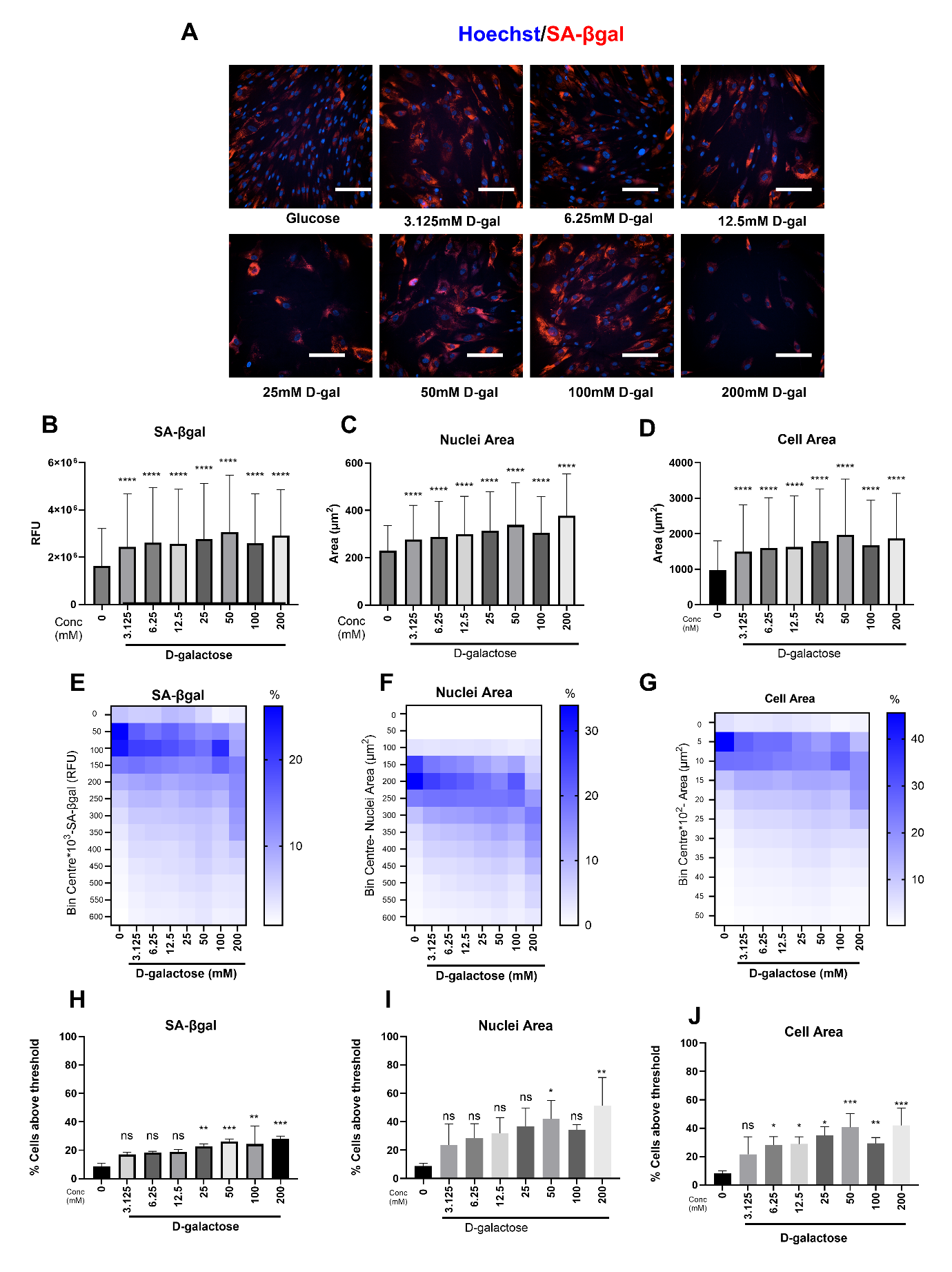


**Supplementary Figure 2. Senescence markers in D-galactose (D-gal)-induced senescence.** (**A**) Representative image of HDFs cultured in glucose-containing media and various concentrations of D-galactose (3.125–200 mM) with nuclei stained with Hoechst (blue) and SA-βgal (red); scale bar: 200 µm. Images were taken using the Opera Phenix™ Plus at 20x magnification. Average total fluorescence intensities of SA-βgal (**B**), average nuclear area (**C**), and cell area (**D**) in HDFs cultured in D-galactose media; error bars represent mean ± standard deviation from one experiment with three replicates. *****p* < 0.0001 (one-way ANOVA with Kruskal-Wallis test compared to the control group). Individual cell-derived heatmaps of SA-βgal (**E**), nuclear area (**F**), and cell area (**G**) of HDFs cultured in D-galactose media. Percentage of cells with SA-βgal fluorescence total intensity (**H**), nuclear area (**I**), and cell area (**J**) above the threshold set for cells cultured in glucose-containing media; ns, not significant (*p* > 0.05), **p* < 0.05, ***p* < 0.01, ****p* < 0.001 (ordinary one-way ANOVA compared to control group).


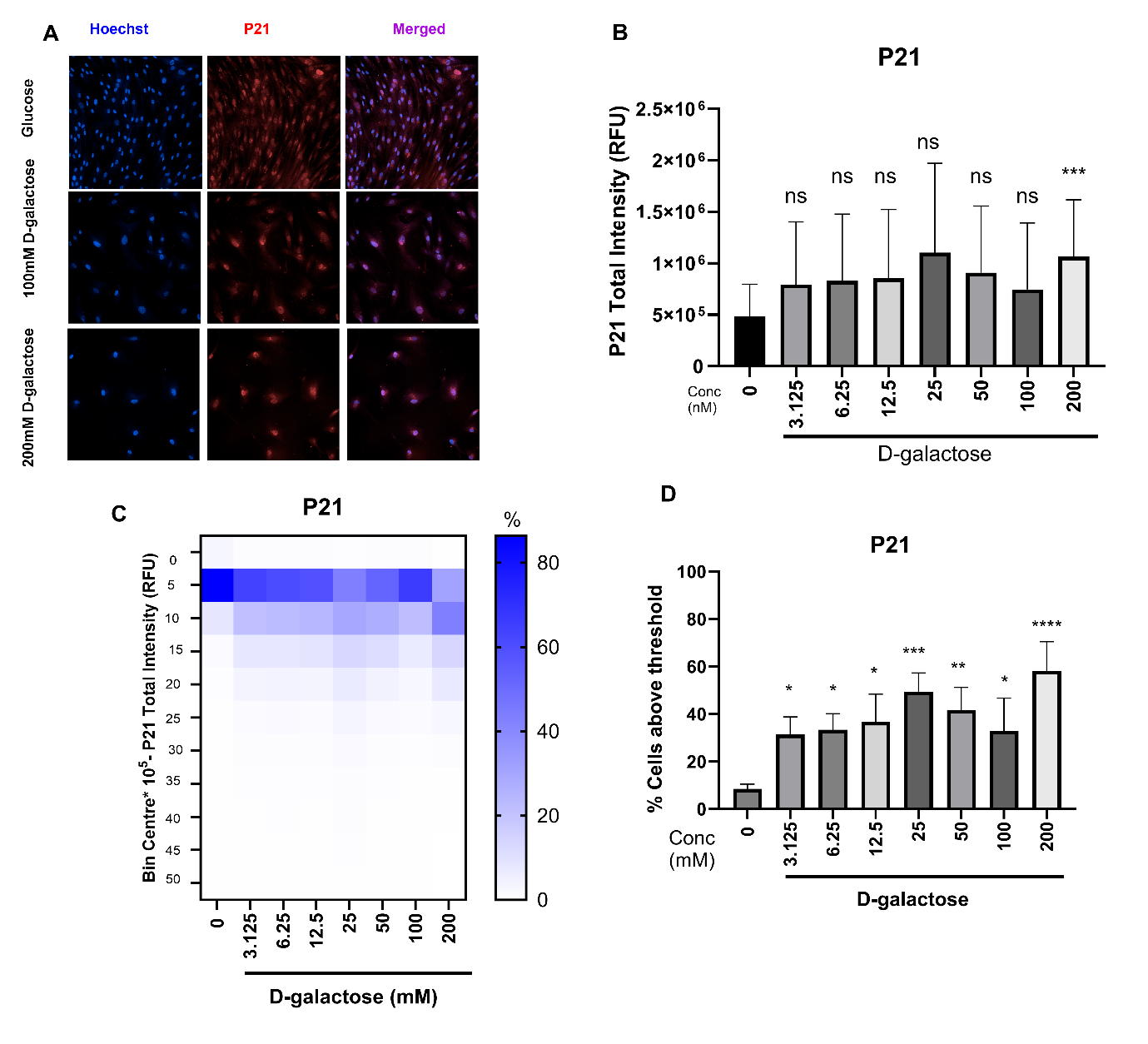


**Supplementary Figure 3. Expression of p21 in D-galactose-induced senescence.** (**A**) Representative images of p21 in HDFs cultured in D-galactose-containing media with nuclei stained with Hoechst (blue), p21 (red), and merged; scale bar: 200 µm. Images were taken using the Opera Phenix™ Plus at 20x magnification. (**B**) Total fluorescence intensity of nuclear p21 in HDFs cultured in D-galactose-containing media; *****p* < 0.0001 (one-way ANOVA with Kruskal-Wallis test compared to the control group). (**C**) Single-cell histogram of total fluorescence intensity of nuclear p21 in HDFs cultured in D-galactose-containing media. (**D**) Percentage of cells positive for p21 expression in HDFs cultured in D-galactose-containing media, assessed using the induction threshold method; **p* < 0.05, ***p* < 0.01, ****p* < 0.001, *****p* < 0.0001 (ordinary one-way ANOVA compared to control group).


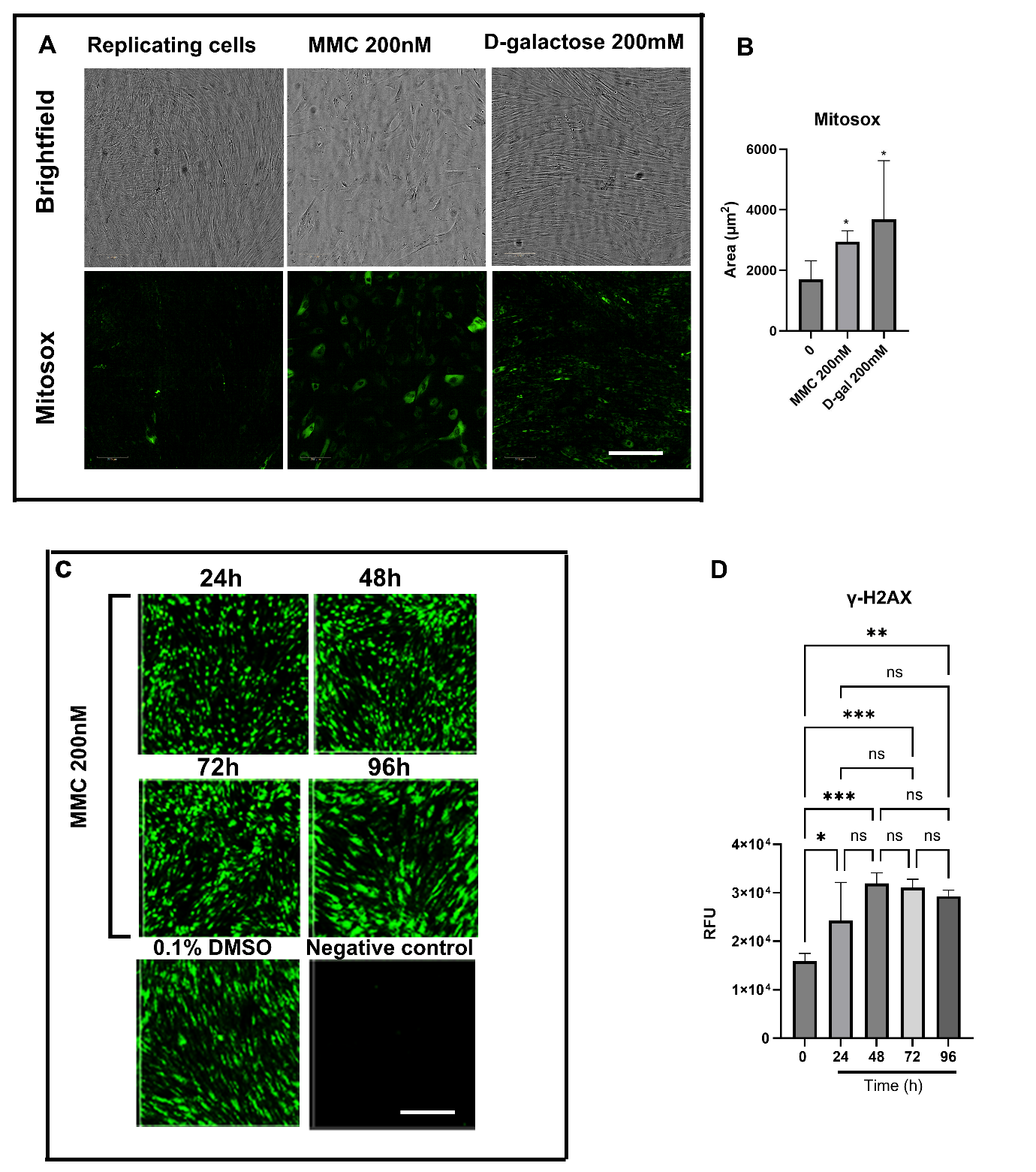


**Supplementary Figure 4. Assessment of mitochondria superoxide production and DNA damage in accelerated senescence models**. Representative images were taken using Opera Phoenix plus ^TM^ at 10X magnification. 0 represents HDFs treated with 0.1% DMSO. **(A)** Representative images of Brightfield and MitoSOX^TM^ green in HDFs treated with either vehicle (0.1% DMSO, MMC 200 nM or D-galactose 200mM, Scale bar: 100µm. **(B)** Percentage area stain of MitoSOX^TM^ green in HDFs treated with either vehicle - 0.1% DMSO, MMC 200 nM or D-galactose 200mM. Data are presented as mean ± SD from three biological replicates; Statistical analysis was performed using simple unpaired t-test either between MMC and 0 group or D-galactose with the 0 group. **(C)** Representative images of nuclear γ-H2AX (green) at timepoint 24,28,72 and 96h in HDFs treated with either vehicle 0.1% DMSO or MMC 200 nM; Scale bar: 1 mm. Data are presented as mean ± SD from three biological replicates; Statistical analysis was performed using one-way ANOVA followed by Tukey’s post hoc test for multiple comparisons between all groups.; (ns- not significant, * *p* < 0.05 ** *p* < 0.01 *** *p*< 0.001 **** *p* < 0.0001).


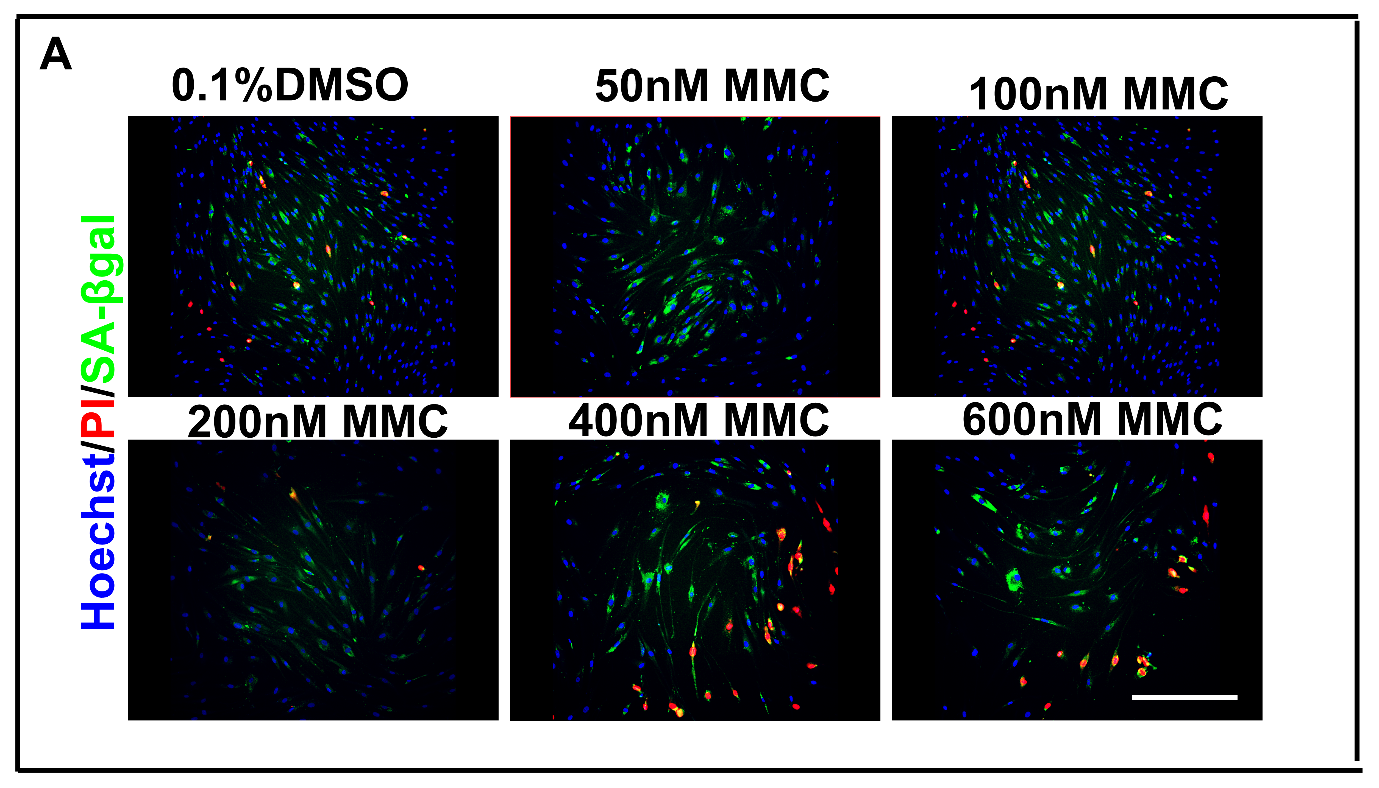


**Supplementary Figure 5.** **Assessment of effect of cell death in accelerated model of cellular senescence.** Representative images were taken using IN-CELL analyser at 10X magnification. **(A)** Merged images of Hoechst (Blue), Propidium iodide (Red) and SA-βgal (green) seven days post treatment with either vehicle (0.1% DMSO) or 50-600nM MMC; Scale bar: 100µm.


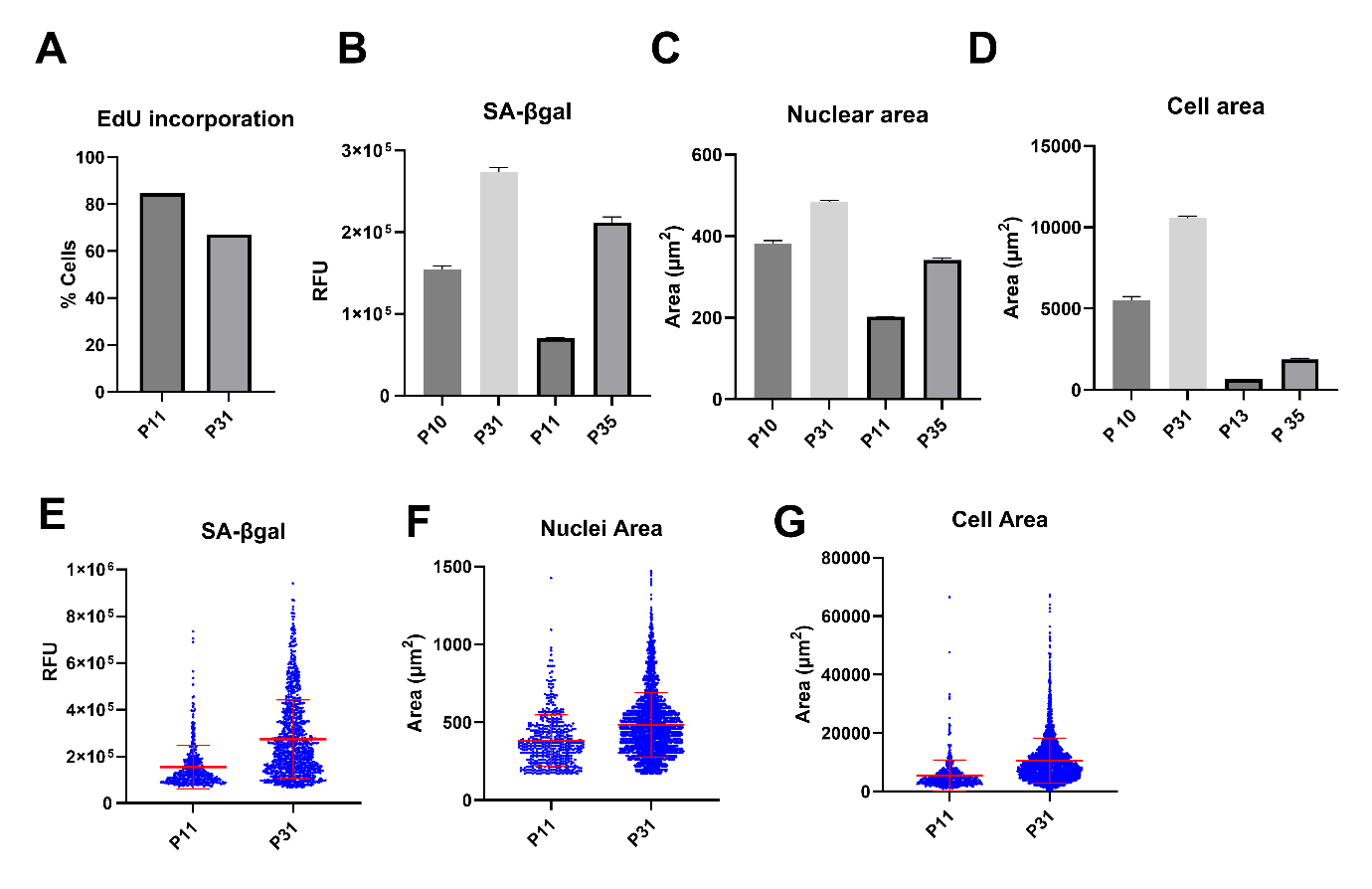


**Supplementary Figure 6.** **Assessment of senescence biomarkers in natural model of cellular senescence.** **(A)** Percentage cells incorporating EdU in early passage (P10) and late passage (P31) HDFs. Average total fluorescence intensity of SA-βgal activity **(B)** along with nuclear **(C)** and cell area **(D)** in in early passage (P10 and P11) and late passage (P31 and P35) HDFs. Single cell data of fluorescence intensity of SA-βgal activity **(E)** along with nuclear **(F)** and cell area **(G)** in in early passage (P10) and late passage (P31) HDFs.


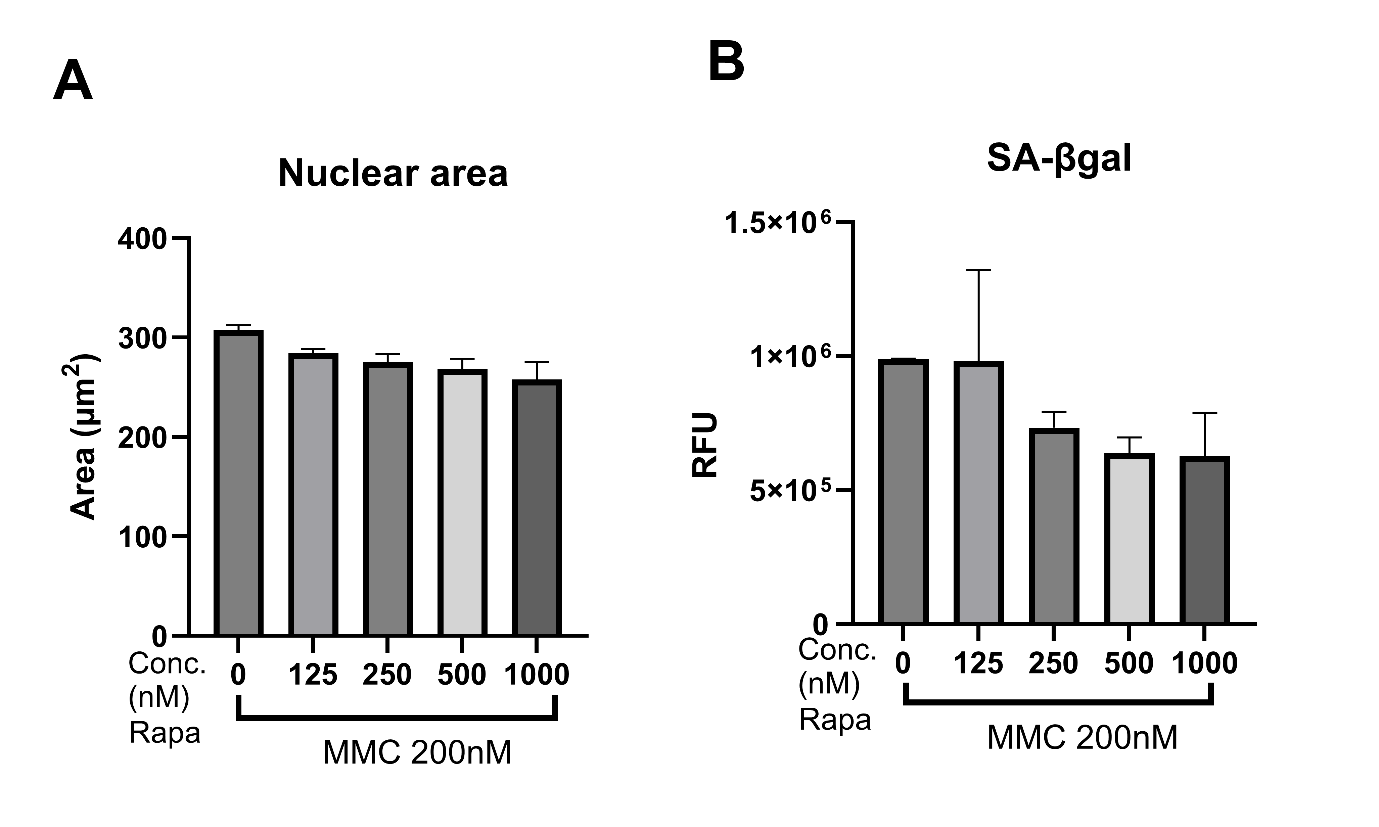


**Supplementary figure 7**. **Concentration response of rapamycin in attenuating senescence biomarkers in accelerated model of senescence.** **(A)** Average nuclear area and total fluorescence intensity SA-βgal enzyme activity **(B)** in HDFs treated with MMC 200 nM and rapamycin 125-1000nM. Data represents Mean±SD from one biological replicate.


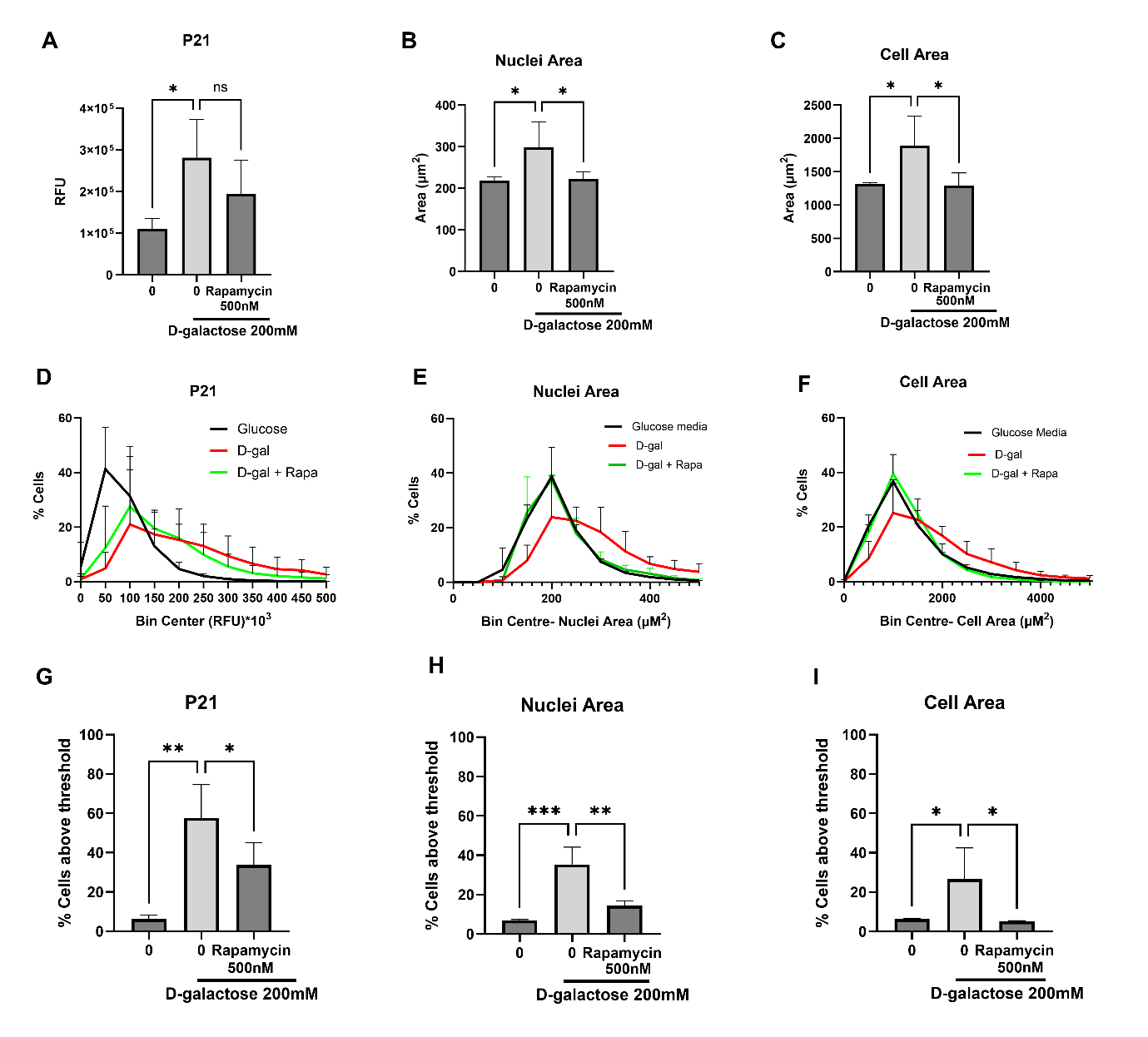


**Supplementary Figure 8. Assessment of senescence biomarkers in D-galactose-induced model of senescence with rapamycin treatment**. Average total fluorescence intensity of P21 (**A**), nuclear area (**B**), and cell area (**C**) in HDFs cultured in either glucose media, 200 mM D-galactose media, or 500 nM rapamycin plus 200 mM D-galactose media. Data represents the mean ± SD from n = 3 biological replicates; * *p* < 0.05, (ordinary one-way ANOVA compared to the 0 (D-galactose media) group). Sub-population analysis of p21 (**D**), nuclear area (**E**), and cell area (F) in HDFs cultured in either glucose media, 200 mM D-galactose media, or 500 nM rapamycin plus 200 mM D-galactose media. Percentage of cells with increased expression of P21 (**G**), nuclear area (**H**), and cell area (**I**), in HDFs cultured in either glucose media, 200 mM D-galactose media, or 500 nM rapamycin plus 200 mM D-galactose media, using the induction threshold method. Data represents the mean ± SD from n = 3 biological replicates; **p* < 0.05, ***p <*0.01, *** *p* < 0.001, (ordinary one-way ANOVA compared to the 0 (D-galactose media) group).


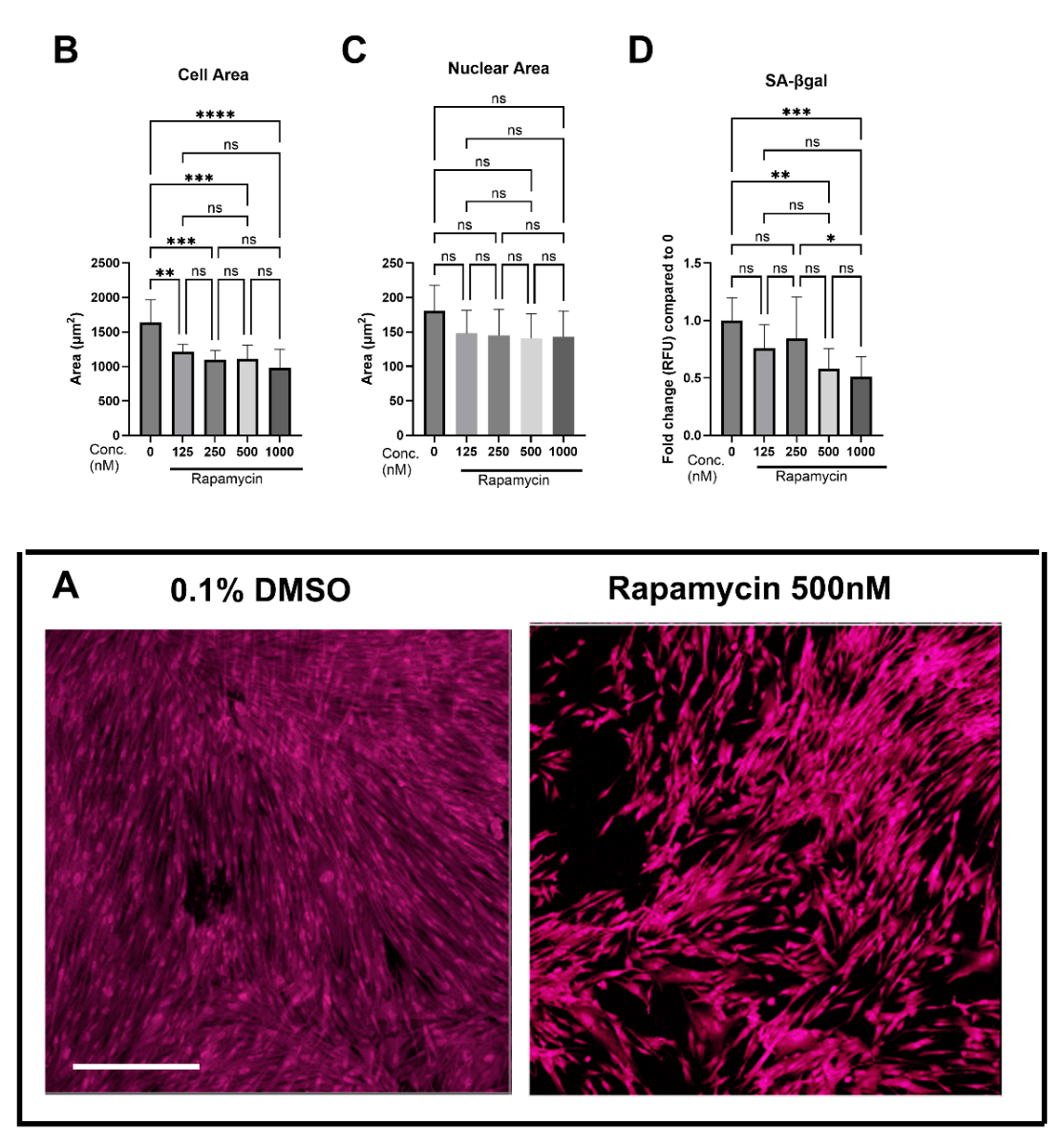


**Supplementary Figure 9. Assessment of the effect of rapamycin on cell area and other senescence biomarkers in normally replicating cells.** Representative images were taken using Opera Phoenix plus^TM^ at 10X magnification. **(A)** Representative images of cell mask (Pink) in proliferating HDFs treated with vehicle (0.1% DMSO) and -500 nM rapamycin; Scale bar: 100µm. Average cell **(B)** and nuclear area **(C)** along with SA-βgal **(D)** in proliferating HDFs treated with vehicle (0.1% DMSO) or 125-1000nM rapamycin. Data are presented as mean ± SD from three biological replicates; Statistical analysis was performed using one-way ANOVA followed by Tukey’s post hoc test for multiple comparisons between all groups.; (ns- not significant, * *p* < 0.05, ** *p* < 0.01, *** *p*< 0.001, **** *p* < 0.0001).

1. **Replicating cells with ABT-263**


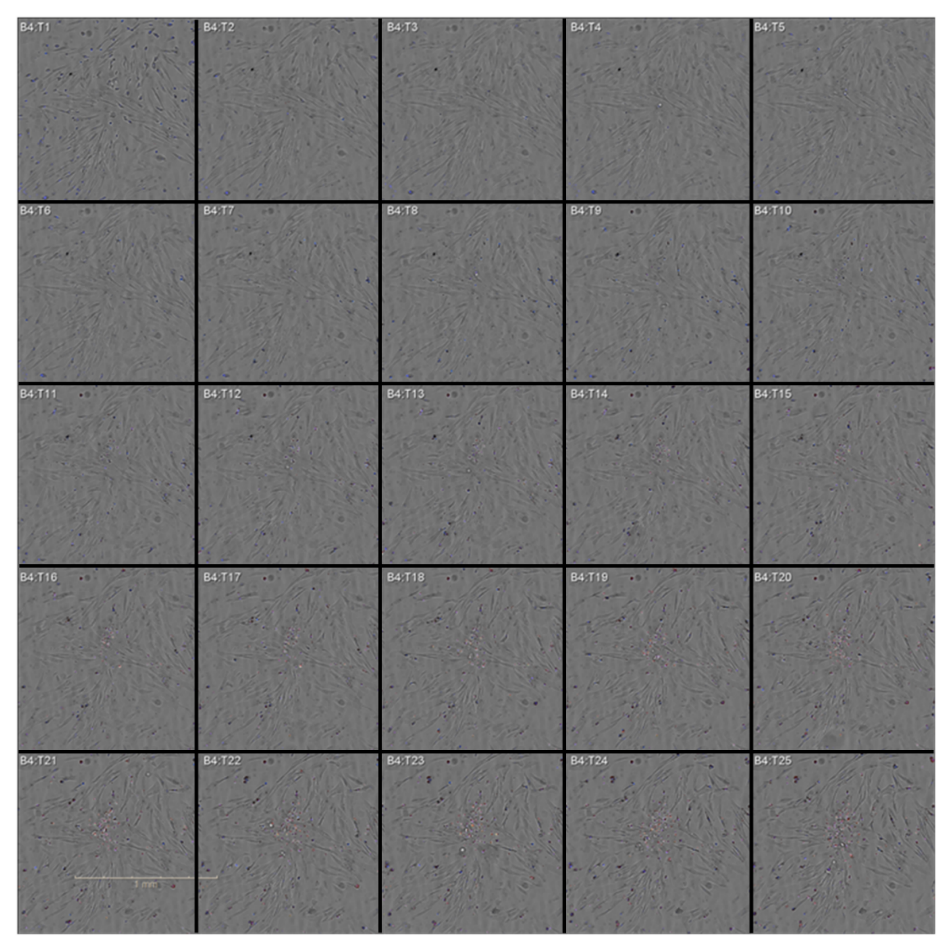


1. **MMC-induced senescent plus ABT-263**


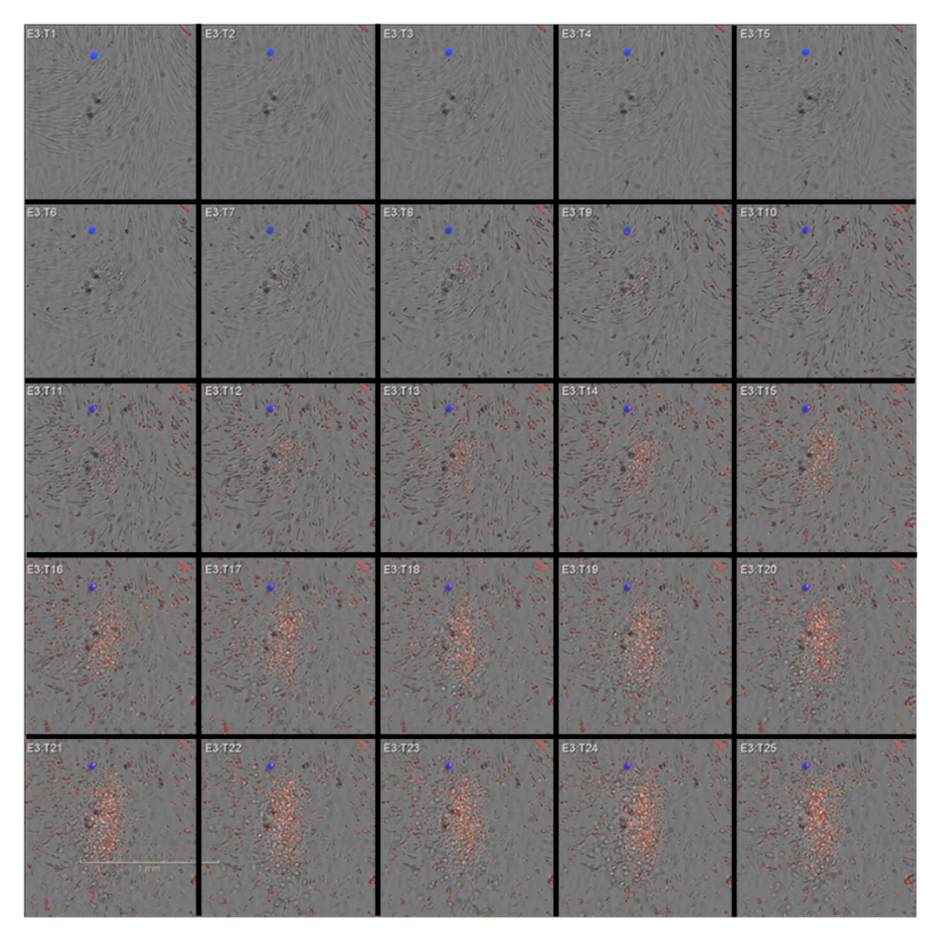


1. **D-galactose induced senescent HDFs plus ABT-263**


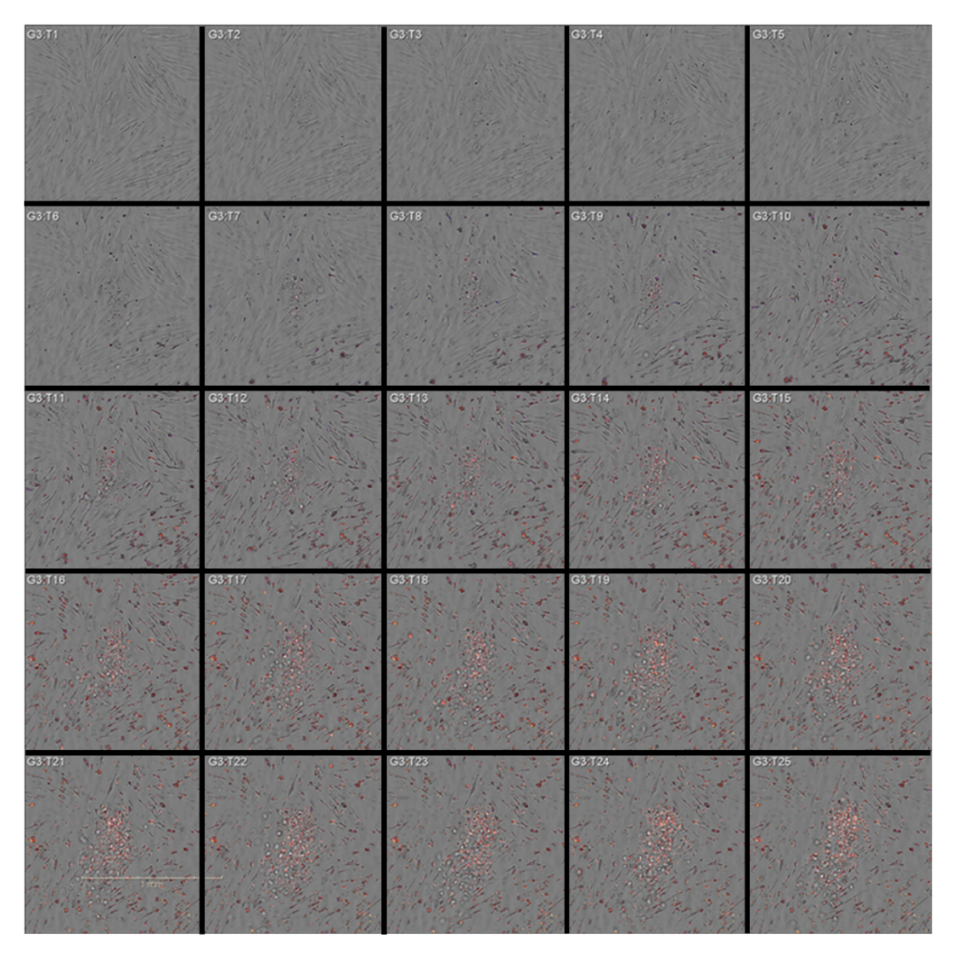


**Supplementary Figure 10. Assessment of the senolytic activity of ABT-263 in replicating cells and in MMC- or D-galactose-induced models of accelerated cellular senescence.** Representative images were taken using Opera Phoenix plus ^TM^ at 10X magnification. Representative images show caspase3/7 activation (green) over 25 timepoints (each interval comprising of 30 minutes (T0-T25) in HDFs treated with either vehicle (0.1% DMSO) **(A)**, MMC 200 nM **(B)** or D-galactose 200mM **(C)**; Scale bar: 1mm. Wells B4, E3, and G3 were selected as representative images for the three experimental groups.
